# De-Novo Design of Antimicrobial Peptides for Plant Protection

**DOI:** 10.1371/journal.pone.0071687

**Published:** 2013-08-12

**Authors:** Benjamin Zeitler, Areli Herrera Diaz, Alexandra Dangel, Martha Thellmann, Helge Meyer, Michael Sattler, Christian Lindermayr

**Affiliations:** 1 Department of Environmental Science, Institute of Biochemical Plant Pathology, Helmholtz Zentrum München, German Research Center for Environmental Health, München-Neuherberg, Germany; 2 Institute of Structural Biology, Helmholtz Zentrum München, German Research Center for Environmental Health, München-Neuherberg, Germany; 3 Department Chemie, Munich Center for Integrated Protein Science at Chair of Biomolecular NMR, Technische Universität München, Garching, Germany; Universidad Pública de Navarra, Spain

## Abstract

This work describes the *de-novo* design of peptides that inhibit a broad range of plant pathogens. Four structurally different groups of peptides were developed that differ in size and position of their charged and hydrophobic clusters and were assayed for their ability to inhibit bacterial growth and fungal spore germination. Several peptides are highly active at concentrations between 0,1 and 1 µg/ml against plant pathogenic bacteria, such as *Pseudomonas syringae*, *Pectobacterium carotovorum*, and *Xanthomonas vesicatoria*. Importantly, no hemolytic activity could be detected for these peptides at concentrations up to 200 µg/ml. Moreover, the peptides are also active after spraying on the plant surface demonstrating a possible way of application. In sum, our designed peptides represent new antimicrobial agents and with the increasing demand for antimicrobial compounds for production of “healthy” food, these peptides might serve as templates for novel antibacterial and antifungal agents.

## Introduction

Higher organisms are continuously exposed to a great variety of pathogens such as viruses, mycoplasma, bacteria, and fungi. To fight these microbes they have developed several defense strategies, including the production of antimicrobial peptides (AMP). AMPs are effective weapons against a wide range of pathogens and are distributed throughout the animal and plant kingdom, suggesting that they are critical for the successful evolution of complex multicellular organisms [1], [2]. Despite their high sequence diversity, AMPs share fundamental structural properties [3] such as short size, positive net charge, hydrophobic nature and clustering of cationic and hydrophobic amino acids within distinct domains of the molecule. Upon contact with pathogen membranes AMPs tend to adopt amphiphilic structures [4]. Because of their cationic and hydrophobic features, antimicrobial peptides interact primarily with negatively charged biomembranes [5], [6]. Many bacterial membranes contain negatively charged components like hydroxylated phospholipids, lipopolysaccharides and teichonic acids and are therefore major targets for AMPs. The hydrophobic regions of the AMPs support incorporation of the peptides into the membranes, leading to pore formation and permeabilization. Several different models have been proposed for peptide insertion, of which the barrel-stave model, the carpet model, and the toroidal-pore model are the most popular ones [6].

In plant protection, bacterial infections are hard to overcome, considering that plant disease control is mainly based on the application of chemical pesticides, which are under strong restrictions and regulatory requirements [7], [8]. However, about 14% of the total loss of all crops produced worldwide are caused by infectious diseases, resulting in a total annual loss of about 220 $ billion per year – not including the 6–12% losses of crop after harvest [9]. Furthermore, microbial organisms often produce toxic compounds, which make food products uneatable or even dangerous for humans and animals. Therefore there is an urgent need for new antimicrobial agents.

AMPs have attracted the interest of researchers for many years. Especially their mode of action, namely targeting fundamental features of microbial cell membranes, is thought to reduce the risk of resistance development in microbial populations – as it happened in the past to every new antibiotic within a few years of its utilization [10] – since this would require a reorganization of the bacterial membranes. However, it has to be mentioned that resistance can be also acquired due to active degradation of the peptides by proteases or due to binding of the peptides to certain cell envelope structures/compounds that decrease effective concentrations.

Nevertheless, the difference in prokaryotic and eukaryotic membrane architecture already imparts selectivity of AMPs for microorganisms and reduces toxic side effects against cells of higher organisms.

In plants several families of antimicrobial peptides have been identified, such as thionins, defensins, lipid transfer proteins, hevein-and knottin-like proteins and snakins, differing in structure, size and cysteine content [11]. The role of antimicrobial peptides in defense is well established and their use in agriculture was already proposed when they were first discovered. Especially antimicrobial peptides from animals were analysed for their plant protecting potential. Magainin (frog), cecropin (silkmoth) and modified or chimeric forms of these two peptides were mainly used in *in-vitro* or *ex-vivo* (detached leaves or fruits) studies against plant pathogens [12]–[16]. However, since the cationic and hydrophobic characteristics of the antimicrobial peptides determine their mode of action, direct modification of these features allows the rational design of new AMPs.

Here, we present the design of a novel set of antimicrobial peptides harbouring different structural and chemical properties, and depict their possible use in plant protection. Several of our designed peptides were highly toxic for a wide range of bacterial and fungal plant pathogens, e.g. *Pseudomonas corrugata*, *Xanthomonas vesicatoria*, and *Cladosporium herbarum* at concentrations below 1 µg/ml, whereas no toxic effects against human cells or plant protoplasts were observed at these concentrations. Altogether, more than 60 peptides were designed and analyzed for their potential use as plant protecting agents in *in-vitro* inhibition assays. Furthermore, spraying the designed peptides on the surface of infected leaves demonstrated their antimicrobial activity directly on plants and displays a way of practical application.

## Results

### Design of Antimicrobial Peptides

**Figure 1:**
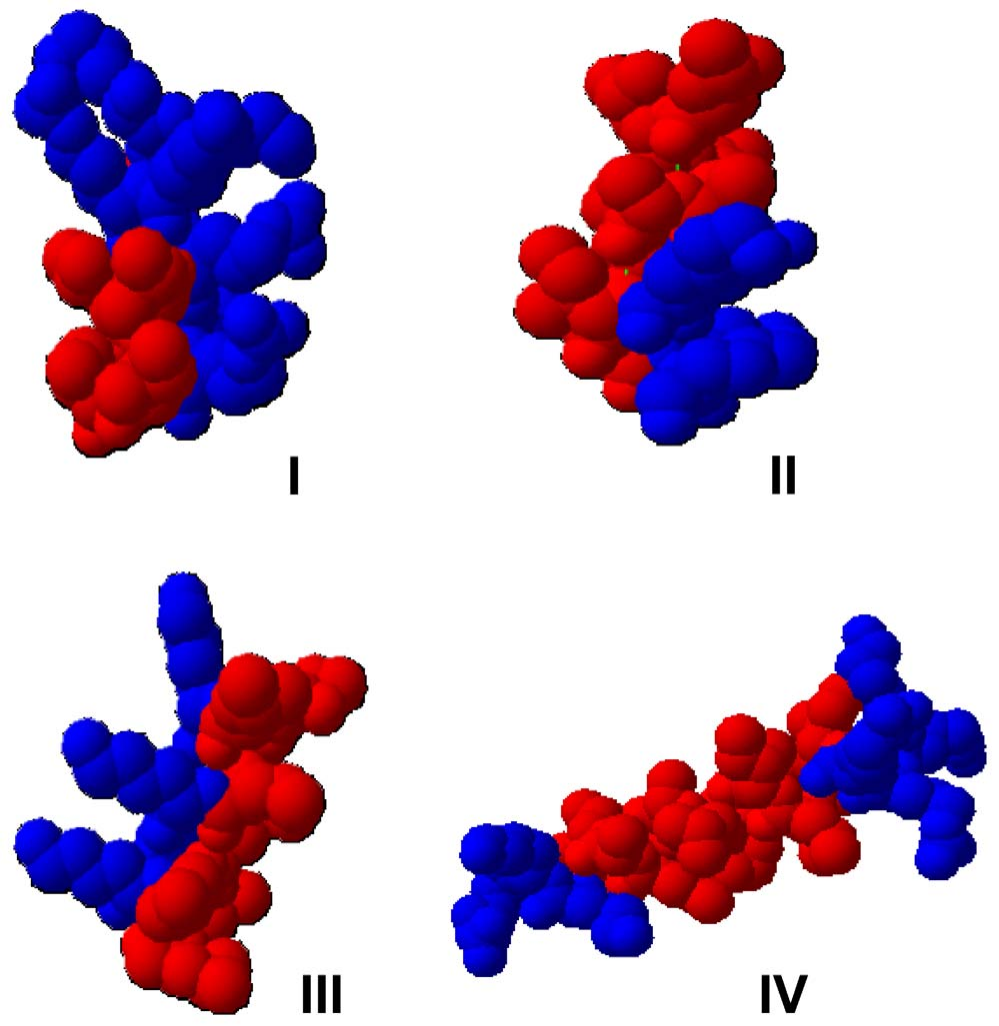
3D-model showing the four lead structures of designed peptides. Four leading structures (I–IV) were designed differing in charge, hydrophobicity, location and size of the hydrophobic and charged cluster. Amino acids with hydrophobic and positively charged side chains are marked in red and blue, respectively. Three-dimensional modelling was performed using the SWISS PdbViewer (ExPASy website. Available: http://http://spdbv.vital-it.ch/. Accessed 2013 July 12) with the helical structures of IsCT derivative (PDB code: 1T52) and magainin II (PDB code: 2MAG) as template.

Several natural occurring peptides show antimicrobial activity *in-vitro* against human, animal, and plant pathogens [8], [17]–[20]. We tested some natural peptides in a microdilution assay for their potential use in plant protection. Besides the two human peptides cathepsin G and histatin 5 we analysed protegrin I (pig), indolicidin (cattle) and magainin II (frog). However, only protegrin I, indolicidin and magainin II were active against some of the tested plant pathogens at concentrations of 1 to 8 µg/ml (Table S1). Unfortunately, natural AMPs often exhibit high hemolytic activity (10–100 µg/ml), making a commercial application problematic [2], [14], [21]. Based on the typical features of natural occurring AMPs we designed a set of sixteen peptides. Since many natural AMPs have a helical structure [5], [22]–[24], this conformation was used as skeletal backbone for the peptides. Furthermore, a typical feature of AMPs is their amphipathicity provided by clusters of hydrophobic and positively charged amino acids. A positive net charge of the designed peptides was guaranteed by using arginine, lysine, and histidine residues in the sequence. Leucine, isoleucine, valine, phenylalanine, alanine, methionine, glycine, serine, and threonine residues were used to generate hydrophobic regions. A helical structure of the peptides was ensured by inserting strong helix-forming amino acids, such as leucine and alanine. We selected a derivative of the scorpion-derived antimicrobial peptide IsCT [25] (12 AA peptides) and the frog-derived peptide magainin II [26] (for 20 AA peptides) as templates. The mutation tool of the SWISS-Pdbviewer software [27] was used to modify the template molecules and to design new peptides. The software enables to see directly a structural model of the designed peptides. To investigate, whether a distinct structural pattern is particular important for antimicrobial activity, four leading structures (group I–IV) were designed, each containing four peptides differing in charge, hydrophobicity, location and size of the hydrophobic and charged clusters (Table 1 and Figure 1). A detailed description of the designing strategy can be found in the supplement (Figure S1).

**Table 1:** Sequences and structural-chemical properties of peptides of the 1^st^ generation.

Peptide	Amino acid sequence		Charge at pH 7^a^	pI^b^	H [peptide]^b^	H [cluster]^c^	Secondary structure^d^
**Group I**							
** SP1**	RKKRLKLLKRLV-NH_2_	+6.76		12.31	−0.808	2.66	–HHHHHHHH–
** SP2**	RKRAARLLKRLV-NH_2_	+5.76		12.48	−0.550	2.63	–HHHHHHHH–
** SP3**	RKRFARFAKRAV-NH_2_	+5.76		12.48	−0.883	2.26	–HHHHHHH–
** SP4**	KKKAARALKRAL-NH_2_	+5.76		12.03	−0.817	2.06	–HHHHHHHH–
**Group II**							
** SP5**	LLIAAFKKLVKK-NH_2_	+3.76		10.48	0.908	3.97	–HHHHHHHHH–
** SP6**	ALAHFLKKAIKK-NH_2_	+3.84		10.48	0.125	3.15	no prediction possible
** SP7**	LLIKFLKRFIKH-NH_2_	+3.84		11.26	0.550	4.27	–HHHHHHHHH–
** SP8**	LLIRAAKKFIKK-NH_2_	+4.76		11.33	0.242	3.63	HHHHHHHHHH–
**Group III**							
** SP9**	LLKALKKLLKKLL-NH_2_	+4.76		10.60	0.685	3.96	HHHHHHHHHHHH–
** SP10**	LRFLKKILKHLF-NH_2_	+3.84		11.26	0.492	4.07	–HHHHHHHHH–
** SP11**	LRALAKALKHKL-NH_2_	+3.84		11.26	0.100	2.87	–HHHHHHHHH–
** SP12**	LKALRKALKHLA-NH_2_	+3.84		11.26	0.100	2.87	–HHHHHHHHH–
**Group IV**							
** SP13**	KRRLIARILRLAARALVKKR-NH_2_	+8.76		12.70	−0.155	5.12	–HHHHHHHHHHHHHH–
** SP14**	KRKLTLVFGVMAGVIGTKKR-NH_2_	+5.76		12.30	0.200	4.69	–EEEHH-EE-E–
** SP15**	KRKLIFLAAFLAALALFKKR-NH_2_	+5.76		12.03	0.815	6.46	–HHHHHHHHHHHHHH–
** SP16**	KRRLAAFRAFRGALKSVLKK-NH_2_	+7.76		12.48	−0.320	4.25	–HHHHHHHHHHHHHHH–

a Estimated using the program Vector NTI 9.1 (Invitrogen).

b Calculated using ProtParam tool (http://www.expasy.org/tools/protparam.html, [84]), H [peptide], grand average hydrophobicity of full peptide.

c H [cluster], hydrophobicity of the hydrophobic cluster of the peptides with the calculation based on the hydrophobicity scales for amino acids (Eisenberg, 1984).

d Secondary structure prediction according to NNPREDICT; H, helix; E, strand; -, no prediction [31]. pI, isoelectric point.

The amino acid sequences were analysed against an AMP database to ensure that they are differ from sequences of already known AMPs [28]. Hydrophobicity was calculated based on the hydrophobicity scale for amino acids [29] and pI values were calculated using the ExPASy ProtParam tool [30]. The helical structure was predicted using NNPREDICT program for protein secondary structure prediction [31]. Peptides of group I consist of a dominant charged cluster and a small hydrophobic region (SP1–SP4). Group II (SP5–SP8) contains peptides with a dominant hydrophobic cluster and a small charged region. In all peptides of group III (SP9–SP12) the hydrophobic and the charged regions have the same size and are separated lengthwise of the molecule. In peptides of group IV (SP13–SP16) the charged regions are located at the N- and C-termini, which are separated by a central hydrophobic cluster. In peptides SP13 and SP16 the charged N-terminal and C-terminal parts are connected by a charged bar.

### Antimicrobial Activities Against Plant Pathogens

Antimicrobial activity of the designed peptides against the plant pathogenic fungi Botrytis cinerea, Alternaria alternata and Cladosporium herbarum, the bacterial plant pathogens Clavibacter michiganensis ssp. michiganensis, Xanthomonas vesicatoria, Pectobacterium carotovorum ssp. carotovorum, and three different Pseudomonas spp. was determined in a microdilution assay. Generally, the designed peptides displayed lower activities against fungi than against plant bacteria (Table 2). B. cinerea and A. alternata were not inhibited by any of the peptides at concentrations below 100 µg/ml. However, C. herbarum was sensitive to most of the tested peptides at concentrations of 10 to 40 µg/ml, whereas only SP5, SP6, and SP14 required concentrations up to 100 µg/ml for inhibition.

**Table 2:** Antimicrobial activities (MIC) of designed first generation peptides against plant pathogens and hemolytic activities.

Organism	SP1	SP2	SP3	SP4	SP5	SP6	SP7	SP8
**Fungi**								
*Botrytis cinerea*	>100	>100	>100	>100	>100	>100	>100	>100
*Alternaria alternata*	>100	>100	>100	>100	>100	>100	>100	>100
*Cladosporium herbarum*	10	20	40	40	100	100	20	40
**Bacteria**								
*Clavibacter michiganensis ssp. michiganensis*	1	2	20	40	20	>40	5	>40
*Pectobacterium carotovorum ssp. carotovorum*	>40	>40	>40	>40	>40	>40	>40	>40
*Xanthomonas vesicatoria*	2	2	>40	>40	40	40	5	>40
*Pseudomonas syringae pv. tomato*	1	2	5	10	>40	10	5	>40
*Pseudomonas syringae pv. syringae*	2	2	>40	>40	40	5	10	>40
*Pseudomonas corrugata*	2	5	>40	>40	>40	20	10	>40
Hemolytic activity^a^	>200	>200	>200	>200	>200	>200	>200	>200
	**SP9**	**SP10**	**SP11**	**SP12**	**SP13**	**SP14**	**SP15**	**SP16**
**Fungi**								
*Botrytis cinerea*	>100	>100	>100	>100	>100	>100	>100	>100
*Alternaria alternata*	>100	>100	>100	>100	>100	>100	>100	>100
*Cladosporium herbarum*	40	10	20	20	20	100	40	20
**Bacteria**								
*Clavibacter michiganensis ssp. michiganensis*	10	10	>40	>40	2.5	20	2.5	5
*Pectobacterium carotovorum ssp. carotovorum*	>40	20	>40	>40	>40	>40	>40	>40
*Xanthomonas vesicatoria*	5	5	>40	20	2.5	>40	20	5
*Pseudomonas syringae pv. tomato*	10	10	5	2.5	2.5	40	40	5
*Pseudomonas syringae pv. syringae*	10	10	>40	10	2.5	>40	>40	5
*Pseudomonas corrugata*	>40	20	10	>40	2.5	>40	>40	2.5
Hemolytic activity^a^	50	100	>200	>200	100	>200	<20	>200

a Shown are the peptide concentrations (µg/ml) leading to 25% hemoglobin release from human blood cells.

>200 describes a slight hemolytic activity at 200 µg/ml but still below the above mentioned threshold.

With regard to the effect on phytopathogenic bacteria, SP1, SP2, SP7, SP13, and SP16 were the most active peptides against the gram-positive *C. michiganensis* as well as the gram-negative *X. vesicatoria* and *Pseudomonas spp*. Especially SP1 and SP2 effectively inhibited the majority of the tested phytopathogenic bacteria at concentrations of 1 to 5 µg/ml. In contrast, *P. carotovorum* was affected only by SP10 (20 µg/ml), while the other peptides showed no activity against these bacteria. Interestingly, in group IV only peptides which contain charged amino acids within the hydrophobic part of the molecules (SP13 and SP16) inhibited bacterial growth.

Since humans would be exposed to these peptides during agricultural applications, low toxicity of AMPs against human cells is desirable. Therefore, the hemolytic activity of the designed peptides was tested. Human red blood cells were incubated with 0, 20, 50, 100 and 200 µg/ml peptide and the hemoglobin release of human erythrocytes was determined. The natural peptide Protegrin I was included as control. Treatment of blood cells with 50 µg/ml of this peptide resulted in significant hemoglobin release (25%). In contrast, most of the designed peptides were not lytic for erythrocytes at concentrations up to 200 µg/ml. However, SP9, SP10, SP13, SP15 showed obvious hemolytic activities at concentrations below 200 µg/ml (Table 2). Especially SP15, which is poorly active against phytopathogens, displayed high hemolytic activity.

As already mentioned, the designed peptides exhibited quite low activity against the tested pathogenic fungi. This could be due to secreted proteases, which are able to degrade the peptides. Since proteins and peptides containing D-amino acids are often more stable against proteolytic decomposition [32], [33], various L-amino acids of the peptides SP1, SP7, SP10, and SP13 were replaced with D-amino acids (Table S2). This modification increased the activity against fungi, whereas the activity against various bacteria was slightly decreased for SP1-D and increased for SP7-D and SP10-D (Table S3). The antibacterial activity of SP13-D is similar to the activity of SP13. Interestingly, the hemolytic activity of SP10-D and SP13-D was lower than for the corresponding L-amino acid peptides (Table S3).

Peptides from three different groups were selected as lead structures to develop a second generation of peptides by directed exchange of distinct amino acids. SP1 (group I) was selected, since it is the most active one of all designed peptides. SP10 (group III) was selected, since it is the only peptide active against *P. carotovorum*. This bacterium is related to *Erwinia amylovora* - a very dangerous pathogen for plants of the sub-family Pomoideae (e. g. apple and pear trees). Finally, the best peptide of group IV (SP13) was selected. The design of the 2^nd^ generation aimed to get peptides with higher antimicrobial activity and lower hemolytic activity than the corresponding lead structures.

**Figure 2:**
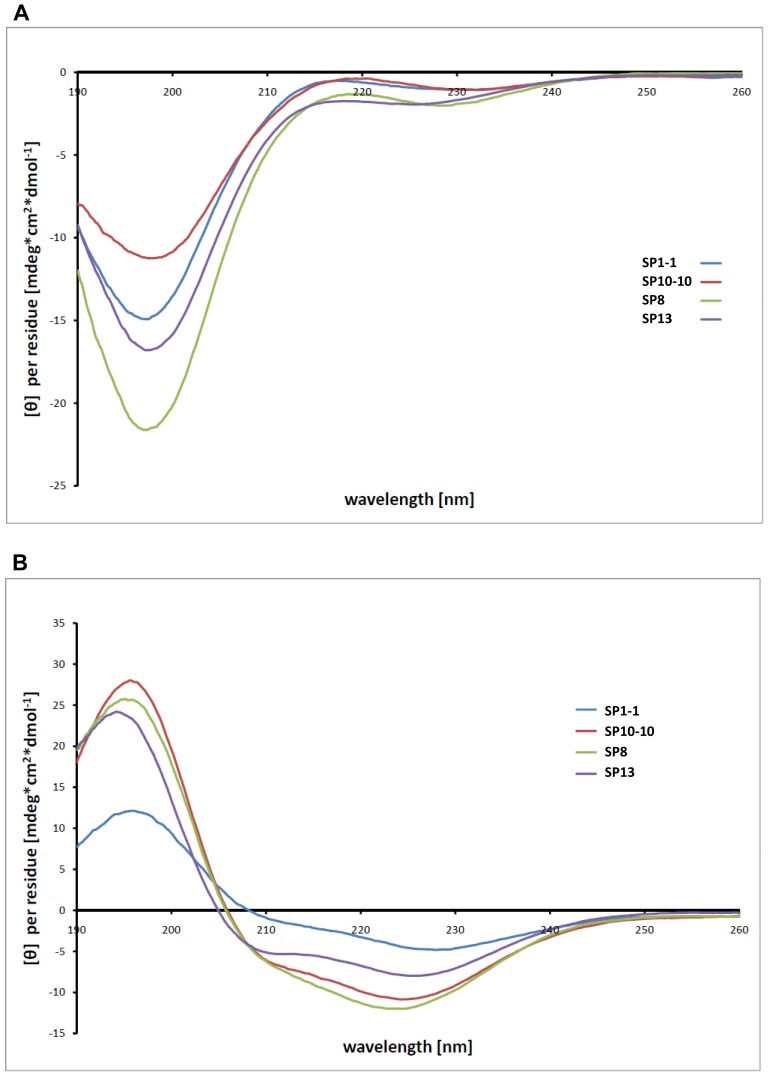
Structural analyses of selected peptides in presence of lipophilic compounds. Circular dichroism (CD) spectra of SP1-1, SP8, SP10-10 and SP13. (A) CD spectra of peptides in ddH_2_O reveal that they are random coil. (B) In the presence of 1,2-dimyristoyl-sn-glycero-3-phospho-sn-glycerol (DMPG) micelles the peptides displayed an α-helical structure. Peptide and DMPG concentrations used for the measurements were 130 µM and 1 mM, respectively.

Twenty-two peptides were derived from SP1, eleven from SP10 and fourteen from SP13 (Table 3). Comparison to the AMP database ensured that the amino acid sequences of the designed peptides are different from sequences of already known AMPs [28]. Special features of these peptides highlighting the most important alteration in comparison to the corresponding lead structures are summarized in Table 3. Derived peptides differed in charge and hydrophobicity, while a helical conformation has been predicted for all designed peptides using the NNPREDICT program [31] (Table S4). Exemplary, the secondary structure formation of one peptide of each subgroup (SP1-1, SP8, SP10-10, SP13) was analyzed by circular dichroism (CD) spectroscopy. As it is known that the local environment and the peptide solvent are important factors that determine orientation and secondary structure formation, the conformation of the peptides was analyzed in presence of water or micelles formed by 1,2-dimyristoyl-sn-glycero-3-phospho-sn-glycerol (DMPG). In water the peptides are random coil (Figure 2A). However, in DMPG micelles all analysed peptides adopt a helical conformation (Figure 2B), indicated by the large positive ellipticity at 195 nm and the negative minima at 208 and 222 nm [34], [35]. Furthermore, secondary structure formation was monitored by NMR spectroscopy. The ^1^H and ^1^H, ^13^C HSQC spectra of free SP1-1 peptide and bound to DPC micelles show significant chemical shift differences. Particularly, the shift of ^1^H^α^ resonances to lower and ^13^C^α^ resonances to higher ppm values is indicative of helix formation (Figure S2). In sum, CD as well as NMR analyses demonstrated that the structure predictions are realistic and that the peptides are able to adopt a helical structure, when they get in contact with a lipophilic environment, such as biological membranes.

**Table 3:** Sequences and structural-chemical properties of peptides of the 2^nd^ generation.

Peptide	AA sequence	Charge pH7^a^	pI^b^	H [peptide]^b^	H [cluster]^c^	Special features
**SP1**	RKKRLKLLKRLV-NH_2_	+6.76	12.31	−0.808	2.66	
**SP1-1**	RKKRLKLLKRLL-NH_2_	+6.76	12.31	−0.842	2.65	> L
**SP1-2**	RKKRVKLLKRLV-NH_2_	+6.76	12.04	−0.725	2.67	> V
**SP1-3**	RKKKVKLLKRLV-NH_2_	+6.76	12.04	−0.725	2.67	> VK
**SP1-4**	RKKRLKVVKRLV-NH_2_	+6.76	12.31	−0.742	2.68	> V
**SP1-5**	RKKRLRVVRRLV-NH_2_	+6.76	12.60	0.842	2.68	> RV
**SP1-6**	RKKKLKVVKRLV-NH_2_	+6.76	12.04	−0.692	2.68	> KV
**SP1-7**	RKKKLKIIKRLI-NH_2_	+6.76	12.04	−0.617	3.25	> hydrophob,>KI
**SP1-8**	RKKKIKIIKRLI-NH_2_	+6.76	12.04	−0.558	3.45	> hydrophob,>KI
**SP1-9**	RKKKIKIIKKII-NH_2_	+6.76	11.43	−0.450	3.65	> hydrophob,>KI
**SP1-10**	RKKKAKIIKKII-NH_2_	+6.76	11.43	−0.675	3.17	> hydrophob,>KI
**SP1-11**	RKKKLKFFKRLF-NH_2_	+6.76	12.04	−1.042	2.89	> hydrophob,>KF
**SP1-12**	RKKKFKFFKRLF-NH_2_	+6.76	12.04	−1.125	2.97	> hydrophob,>KF
**SP1-13**	RKKKFKFFKRFF-NH_2_	+6.76	12.04	−1.208	3.05	> hydrophob,>KF
**SP1-14**	RKKKFKIFKRLF-NH_2_	+6.76	12.04	−0.983	3.09	> hydrophob,>KF
**SP1-15**	KRKKLLKRLL-NH_2_	+5.76	12.03	−0.940	2.12	<+charge,<hydrophob,>L
**SP1-16**	KRKKLLKRLI-NH_2_	+5.76	12.03	−0.870	2.32	<+charge,<hydrophob,>L
**SP1-17**	KKKKIIKRLI-NH_2_	+5.76	11.39	−0.670	2.72	<+charge,>hydrophob,>KI
**SP1-18**	RKKRKKLLKRLL-NH_2_	+7.76	12.32	−1.483	2.12	>+charge,<hydrophob,>L
**SP1-19**	RKKRKKLIKRLI-NH_2_	+7.76	12.32	−1.367	2.52	>+charge,<hydrophob,>K
**SP1-20**	RKKRKKLLKRLI-NH_2_	+7.76	12.32	−1.425	2.32	>+charge,<hydrophob,>KL
**SP1-21**	RKKKKKIIKKLI-NH_2_	+7.75	11.47	−1.208	2.72	>+charge,>hydrophob,>KI
**SP1-22**	KKKKKKIIKKII-NH_2_	+7.75	10.85	−1.100	2.92	>+charge,>hydrophob,>KI
**SP10**	LRFLKKILKHLF-NH_2_	+3.84	11.26	0.492	4.07	
**SP10-1**	LRFLKKILKKLF-NH_2_	+4.76	11.33	0.433	4.07	>+charge,>K
**SP10-2**	LRFLKKALKKLF-NH_2_	+4.76	11.33	0.208	3.59	>+charge,<hydrophob,>KA
**SP10-3**	LRFAKKALKKLF-NH_2_	+4.76	11.33	0.042	3.31	>+charge,<hydrophob,>KA
**SP10-4**	LRFIKKILKKLI-NH_2_	+4.76	11.33	0.633	4.33	>+charge,>hydrophob,>KI
**SP10-5**	LRIIKKILKKLI-NH_2_	+4.76	11.33	0.775	4.51	>+charge,>hydrophob,>KI
**SP10-6**	LRIIRRILRRLI-NH_2_	+4.76	12.60	0.575	4.51	>+charge,>hydrophob,>RI
**SP10-7**	LRILRRLLRRLF-NH_2_	+4.76	12.60	0.317	3.99	>+charge,>RL
**SP10-8**	LRFLRRILRRLL-NH_2_	+4.76	12.60	0.158	3.99	>+charge,>RL
**SP10-9**	LRFARRALRRLF-NH_2_	+4.76	12.60	−0.158	3.31	>+charge,<hydrophob,>RA
**SP10-10**	LRKLKKILKKLF-NH_2_	+5.76	11.39	−0.125	3.46	>+charge,<hydrophob,>K
**SP10-11**	LRKAKKIAKKLF-NH_2_	+5.76	11.39	−0.458	2.90	>+charge,<hydrophob,>KA
**SP13**	KRRLIARILRLAARALVKKR-NH_2_	+8.76	12.70	−0.155	5.12	
**SP13-1**	KRRLIARILRLAIRALVKKR-NH_2_	+8.76	12.70	−0.020	5.60	> hydrophob,>I
**SP13-2**	KRRLILRILRLAIRALVKKR-NH_2_	+8.76	12.70	0.080	5.88	> hydrophob,>IL
**SP13-3**	KRRLILRILRLAIRILVKKR-NH_2_	+8.76	12.70	0.215	6.36	> hydrophob,>IL
**SP13-4**	KRRLIFRILKLFFRFLVKKR-NH_2_	+8.76	12.61	0.075	6.56	> hydrophob,>F
**SP13-5**	KRRILIRILKLIIKLILKKR-NH_2_	+8.76	12.49	0.425	7.03	> hydrophob,>KIL
**SP13-6**	KRRKLIKILKLIIKLIRKKR-NH_2_	+10.75	12.49	−0.380	5.77	>+charge,>hydrophob,>KIL
**SP13-7**	KRRKLIKILKLIAKLIRKKR-NH_2_	+10.75	12.49	−0.515	5.29	>+charge,>hydrophob,>KIL
**SP13-8**	KRRKAIKILKLIAKLIRKKR-NH_2_	+10.75	12.49	−0.615	5.01	>+charge,<hydrophob,>KIL
**SP13-9**	KRRKAIKILKLIAKAIRKKR-NH_2_	+10.75	12.49	−0.715	4.73	>+charge,<hydrophob,>KI
**SP13-10**	KRRLALFRAFRLALKSVLKK-NH_2_	+7.76	12.48	−0.010	4.90	<+charge,<hydrophob
**SP13-11**	KRRLALFRLFRLALKLVLKK-NH_2_	+7.76	12.48	0.320	5.97	<+charge,>hydrophob
**SP13-12**	KRRLFLFRLFRLFLRLFLKK-NH_2_	+7.76	12.61	0.320	6.76	<+charge,>hydrophob,>F
**SP13-13**	KRRKLAFRAFRFALKAVLKK-NH_2_	+8.76	12.49	−0.315	4.96	< hydrophob,>F
**SP13-14**	KRRKLAFRLFRLFLKLVLKK-NH_2_	+8.76	12.49	−0.015	5.80	> hydrophob,>FL

a Estimated using the program Vector NTI 9.1 (Invitrogen).

b Calculated using ProtParam tool (http://www.expasy.org/tools/protparam.html, [30]), H [peptide], grand average hydrophobicity of full peptide.

c H [cluster], hydrophobicity of the hydrophobic cluster of the peptides with the calculation based on the hydrophobicity scales for amino acids [29]. pI, isoelectric point. Special features: Important alterations in comparison to the leading structure are highlighted.>increased,<decreased.

The whole set of the 2^nd^ generation was tested against plant pathogenic bacteria and their hemolytic activity was determined. Most of these peptides did not show an increased activity when compared to the corresponding lead structures. However, the activities of the peptides SP1-1, SP10-2, and SP10-10 were clearly enhanced (Table 4). Especially SP10-2 showed an at least 20-fold higher activity against all tested plant bacteria and particularly the growth of *P. carotovorum* was now inhibited by an 80-fold lower concentration compared to SP10. Furthermore, the activity of SP10-10 against *P. corrugata* was increased in a similar range. Interestingly, SP1-1 showed higher activity against *P. carotovorum*, *X. vesicatoria* and two *Pseudomonas spp*., but growth inhibition of *C. michiganensis* and *P. syringae pv. syringae* was reduced. Interestingly, some peptides derived from SP13 were highly hemolytic. Peptide SP13 induced 25% hemolysis at a concentration of 100 µg/ml and hence was in the mid-range of the tested 1^st^ generation peptides. Changing two amino acids (A6L and A13I) without altering charge and pI, resulted in increased hemolytic activity of SP13-2. With this peptide25% hemolysis could already be detected at a concentration of 50 µg/ml. Similar results were observed for SP13-4, SP13-6, SP13-12 and SP13-14 (Table 4) concluding that the overall structure of these peptides (see Figure 1, group IV) might be responsible for their hemolytic activity.

**Table 4:** Antimicrobial activities (MIC) and hemolytic activities of peptides of the 2^nd^ generation.

Bacteria	SP1	SP1-1	SP1-2	SP1-3	SP1-4	SP1-5	SP1-6	SP1-7	SP1-8	SP1-9
*Clavibacter michiganensis* (2d)	1	2.5	10	>10	>10	>10	10	2.5	5.0	2.5
*Pectobacterium carotovorum* (1d)	>40	>10	>10	>10	>10	>10	>10	>10	>10	>10
*Xanthomonas vesicatoria* (2d)	2	0.1	5.0	>10	>10	>10	>10	1.0	2.5	2.5
*Pseudomonas syringae pv. tomato* (1d)	1	>10	5.0	5.0	2.5	1.0	1.0	10	10	5.0
*Pseudomonas syringae pv. syringae* (1d)	2	0.1	2.5	0.25	5.0	>10	5.0	0.1	0.25	0.5
*Pseudomonas corrugata* (2d)	2	<0.1	0.5	2.5	>10	5.0	>10	0.5	2.5	5.0
Hemolytic activity^a^	–	–	–	–	–	–	–	–	–	–
	**SP1-10**	**SP1-11**	**SP1-12**	**SP1-13**	**SP1-14**	**SP1-15**	**SP1-16**	**SP1-17**	**SP1-18**	**SP1-19**
*Clavibacter michiganensis* (2d)	>10	2.5	0.5	0.25	1.0	5.0	>10	>10	5.0	5.0
*Pectobacterium carotovorum* (1d)	>10	>10	>10	>10	>10	>10	>10	>10	>10	>10
*Xanthomonas vesicatoria* (2d)	>10	2.5	0.5	0.5	1.0	5.0	10	>10	5.0	5.0
*Pseudomonas syringae pv. tomato* (1d)	>10	>10	>10	2.5	1.0	1.0	1.0	1.0	1.0	>10
*Pseudomonas syringae pv. syringae* (1d)	1.0	1.0	0.1	0.1	<0.1	0.1	0.1	<0.1	0.1	<0.1
*Pseudomonas corrugata* (2d)	10	0.25	0.25	<0.1	0.5	<0.1	0.5	5.0	0.25	0.5
Hemolytic activity^a^	–	–	–	–	–	–	–	–	–	–
	**SP1-20**	**SP1-21**	**SP1-22**	**SP10**	**SP10-1**	**SP10-2**	**SP10-3**	**SP10-4**	**SP10-5**	**SP10-6**
*Clavibacter michiganensis* (2d)	5.0	>10	>10	10	1.0	0.25	2.5	0.25	0.5	2.5
*Pectobacterium carotovorum* (1d)	>10	>10	>10	20	2.5	1.0	>10	2.5	2.5	2.5
*Xanthomonas vesicatoria* (2d)	10	>10	>10	5	2.5	0.25	2.5	0.1	0.25	1.0
*Pseudomonas syringae pv. tomato* (1d)	10	2.5	1.0	10	1.0	0.5	0.25	1.0	0.25	1.0
*Pseudomonas syringae pv. syringae* (1d)	0.25	0.5	2.5	10	2.5	0.1	0.25	0.25	0.5	2.5
*Pseudomonas corrugata* (2d)	0.5	2.5	>10	20	2.5	0.5	1.0	2.5	0.5	2.5
Hemolytic activity^a^	–	–	–	100	>200	>200	–	>200	–	>200
	**SP10-7**	**SP10-8**	**SP10-9**	**SP10-10**	**SP10-11**	**SP13**	**SP13-1**	**SP13-2**	**SP13-3**	**SP13-4**
*Clavibacter michiganensis* (2d)	5.0	0.5	1.0	0.25	5.0	2.5	0.5	0.25	2.5	1.0
*Pectobacterium carotovorum* (1d)	>10	2.5	2.5	2.5	>10	>40	>10	>10	>10	>10
*Xanthomonas vesicatoria* (2d)	5.0	0.5	1.0	0.1	0.5	2.5	0.5	0.25	2.5	2.5
*Pseudomonas syringae pv. tomato* (1d)	1.0	1.0	0.25	0.5	0.5	2.5	1.0	10	10	>10
*Pseudomonas syringae pv. syringae* (1d)	>10	0.5	0.25	0.25	0.1	2.5	1.0	5.0	>10	10
*Pseudomonas corrugata* (2d)	2.5	0.5	0.5	0.25	0.5	2.5	2.5	5.0	>10	5.0
Hemolytic activity^a^	–	50	–	–	–	100	>200	50	>200	50
	**SP13-5**	**SP13-6**	**SP13-7**	**SP13-8**	**SP13-9**	**SP13-10**	**SP13-11**	**SP13-12**	**SP13-13**	**SP13-14**
*Clavibacter michiganensis* (2d)	2.5	0.1	0.25	0.1	0.25	0.1	2.5	2.5	0.5	0.5
*Pectobacterium carotovorum* (1d)	>10	>10	>10	>10	>10	>10	>10	>10	>10	>10
*Xanthomonas vesicatoria* (2d)	1.0	0.1	0.1	0.1	0.1	0.1	2.5	2.5	0.25	0.25
*Pseudomonas syringae pv. tomato* (1d)	2.5	2.5	10	10	>10	1.0	2.5	5.0	2.5	2.5
*Pseudomonas syringae pv. syringae* (1d)	>10	1.0	>10	>10	>10	>10	>10	>10	0.5	5.0
*Pseudomonas corrugata* (2d)	10	2.5	0.5	0.25	0.25	0.25	>10	10	0.25	2.5
Hemolytic activity^a^	100	20	200	>200	–	200	200	50	>200	50

Values reflect the MIC (µg/ml) after different incubation periods as indicated in brackets after the organism.

a Shown are the peptide concentrations leading to 25% hemoglobin release from human blood cells.

>200 describes a slight hemolytic activity at 200 µg/ml but still below the above mentioned threshold, no value indicates none detectable hemolytic activity up to the highest concentration tested.

### Membrane Activity of Selected Peptides

**Figure 3:**
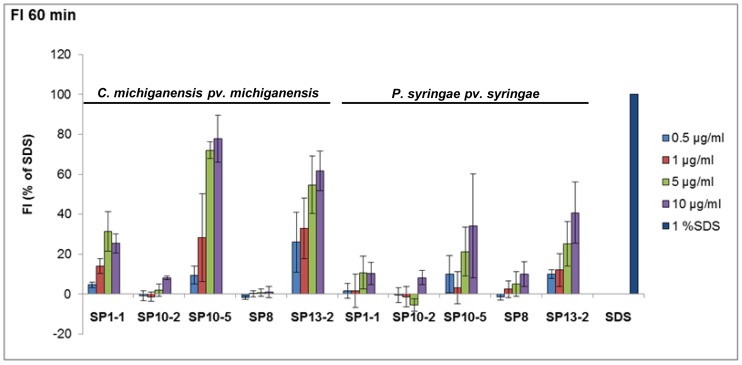
Bacterial membrane depolarization 60 minutes after AMP treatment. Depolarisation of bacterial membranes was determined by loading 5×10^7^ cfu/ml gram-positive *C. michiganensis* or gram-negative *P. syringae pv. syringae* with DiSC3(5) and measuring fluorescence intensity (FI) 60 min after addition of 0.5, 1, 5 or 10 µg/ml peptides (λ_ex_: 622 nm λ_em_: 670 nm). 1% Sodium dodecyl sulfate (SDS) and SP8 were used as positive and negative control, respectively. Shown are mean values ± standard error of the mean of three independent measurements normalized against FI after buffer treatment.

Because of their physico-chemical features most of the antimicrobial peptides are supposed to interact with bacterial membranes. To investigate the ability of the synthetic peptides to disturb membrane homeostasis the membrane potential was analysed with the membrane potential sensitive fluorescent dye DiSC3(5). This cationic dye accumulates on hyperpolarized membranes and is translocated into the lipid bilayer, where it is quenched in response to an intact membrane potential [36]. Under depolarizing condition, the dye is released into the medium and the increasing fluorescence can be measured. Two different strains of phytopathogenic bacteria, one gram-positive (*C. michiganenis*) and one gram-negative strain (*P. syringae* pv. *syringae*) were loaded with the fluorescent dye. Afterwards, the bacteria were treated with 0.5, 1, 5, 10 µg/ml of at least one representative of each group (SP1-1, SP10-2, SP10-5, SP8 or SP13-2). SP1-1, SP10-2, SP10-5, and SP13-2 were selected for these analyses because of their good antibacterial activity (Table 4). Sodium dodecyl sulfate was used as positive control. SP8, a peptide with no antimicrobial activity at the applied concentration, was used as negative control. A summary of all treatments is given in Figure 3. SP10-2 and SP8 showed no or only very low depolarizing activity in both tested bacteria strains, whereas SP10-5 and SP13-2 resulted in significant membrane potential changes in *C. michiganensis* and *P. syringae* pv. *syringae*. Only moderate depolarizing activity was observed for SP1-1. In general, changes of the membrane potential were lower for *P. syringae* pv. *syringae* in comparison to *C. michiganensis*.

### Plant Protecting Properties of Designed Peptides

**Figure 4:**
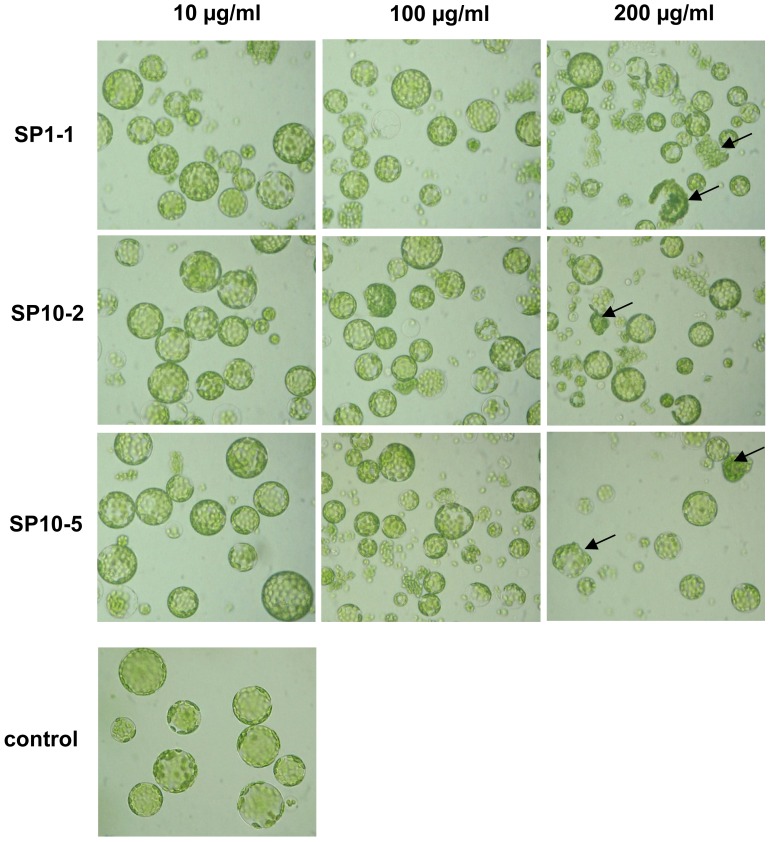
Effect of designed antimicrobial peptides on the viability of Arabidopsis mesophyll protoplasts *in-vitro*. Protoplasts were incubated for 1 h with different concentrations of SP1-1, SP10-2 and SP10-5 and analysed with a microscope (x 200). Cells with spherical shape without any sign of cytoplasmic degradation were defined as viable. A change of the cell shape, chloroplast release and/or agglomeration of protoplasts indicates a toxic effect of the peptides on plant cells (arrows).

To consider a possible practical application the peptides have been tested directly in the plant system. Since many peptides of group IV have a high hemolytic activity, we selected peptides of group I and group III for further studies. The most promising candidates are derivatives of SP1 (SP1-1) and SP10 (SP10-2 and SP10-5). These peptides were highly active against a broad spectrum of bacteria, but showed low hemolytic activity. First, we analysed the toxicity of these peptides for plant cells by incubation with plant protoplast (Figure 4). All three peptides showed a very low phytotoxicity to plant protoplasts and therefore seem to be well suited as a plant protecting agent (as comparison see phytotoxicity of SP15, Figure S3). Hints to cell death are loss of spherical shape, chloroplast release and agglomeration of protoplasts. Such effects could be observed only at concentrations of 200 µg/ml.

**Figure 5:**
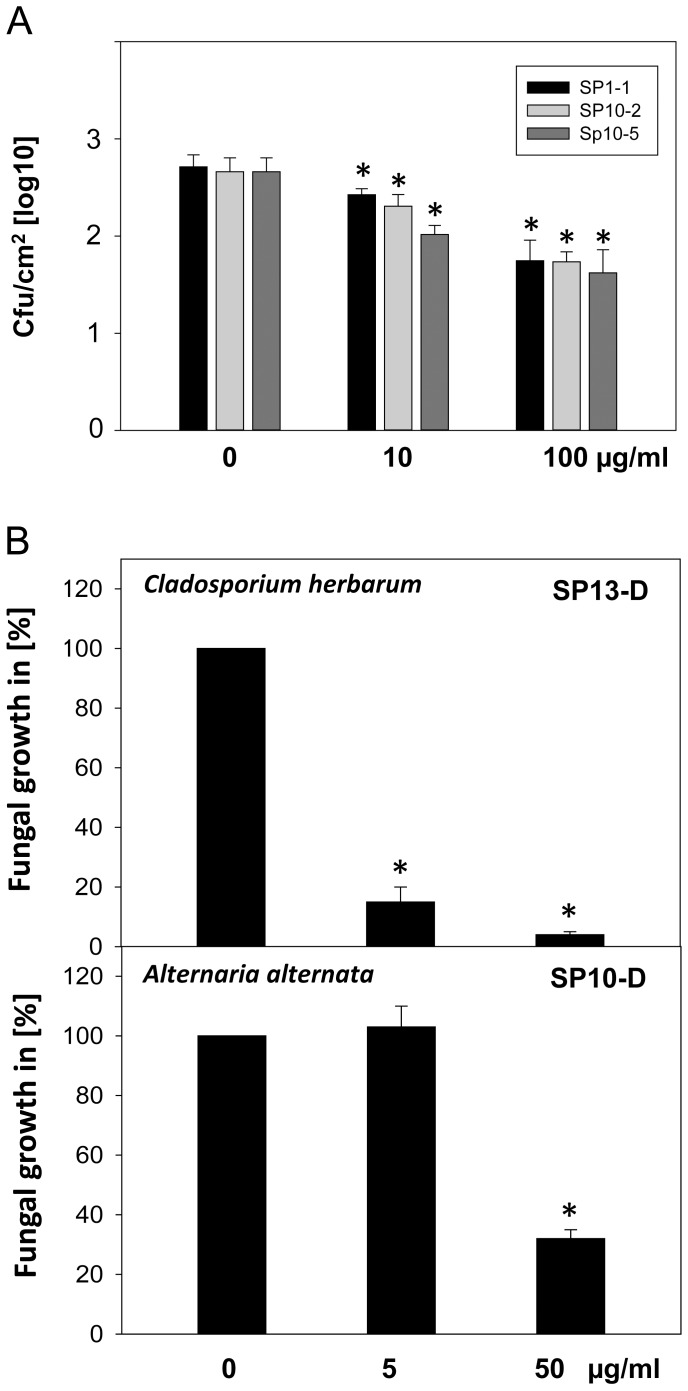
Growth inhibition of phytopathogens on tomato leaves. Tomato leaves were inoculated with (A) virulent *P. syringae pv. tomato* DC3000 (10^7^ CFU/ml) or (B) spores of *A. alternata* or *C. herbarum* (10^4^ spores/ml). Afterwards different concentrations of antimicrobial peptides were sprayed onto the leaves. Bacterial growth was monitored 30 min after peptide treatment by determining colony-forming units per defined leaf area. Fungal growth was analysed 48 h after peptide treatment by quantification of fungal DNA content in the leave tissue. Fungal growth on leaves treated with peptide dilution buffer was set to 100%. Values represent the mean of at least three biological replicates ± standard error of the mean. *indicates significantly lower than the control treatment, *P*<0.05.

To demonstrate that the designed peptides are able to inhibit growth of phytopathogenic microorgansims on the plant surface tomato leaves were inoculated with the virulent *P. syringae pv. tomato* DC3000, the cause of bacterial speck disease. To simulate the natural inoculation, a suspension of *P. syringae* pv. *tomato* DC3000 (10^7^ cfu/ml) was sprayed onto the tomato leaf surface. After 30 min peptides (SP1-1, SP10-2 or SP10-5) were applied onto the inoculated leaves. The most reasonable form of application for plant protection is spraying the peptides on the plant surface as it is done with most of the plant protecting agents. After 30 min of incubation bacterial growth was analysed (Figure 5A). Treatments with 10 µg/ml of SP1-1, SP10-2 and SP10-5 reduced bacterial growth significantly. Peptide concentrations up to 100 µg/ml resulted in growth inhibition of 90% for all tested peptides. In a similar experiment the activity of two peptides (SP10-D and SP13-D) were tested against phytopathogenic fungi. The two peptides displayed no phytotoxicity at concentrations up to 100 µg/ml (Figure S3). Tomato leaves were inoculated with spores (10^4^ spores/ml) of *A. alternata* or *C. herbarum* and after 22 h the antimicrobial peptides were sprayed onto the leaves in different concentrations. One and two days later fungal growth was determined by RT-PCR using specific primers for each fungus (Figure 5B and Figure S4). More than 80% growth inhibition could be observed for *C. herbarum* after treatment with 5 µg/ml SP13-D and around 70% growth inhibition was detected for *A. alternata* after treatment with 50 µg/ml SP10-D (Figure 5B). RT-PCR is a well-established and often used to detect and quantify microorganisms in plants, soil and food [37]–[39]. However, it is important to mention that this method cannot distinguish viable from non-viable spores/fungi.

**Figure 6:**
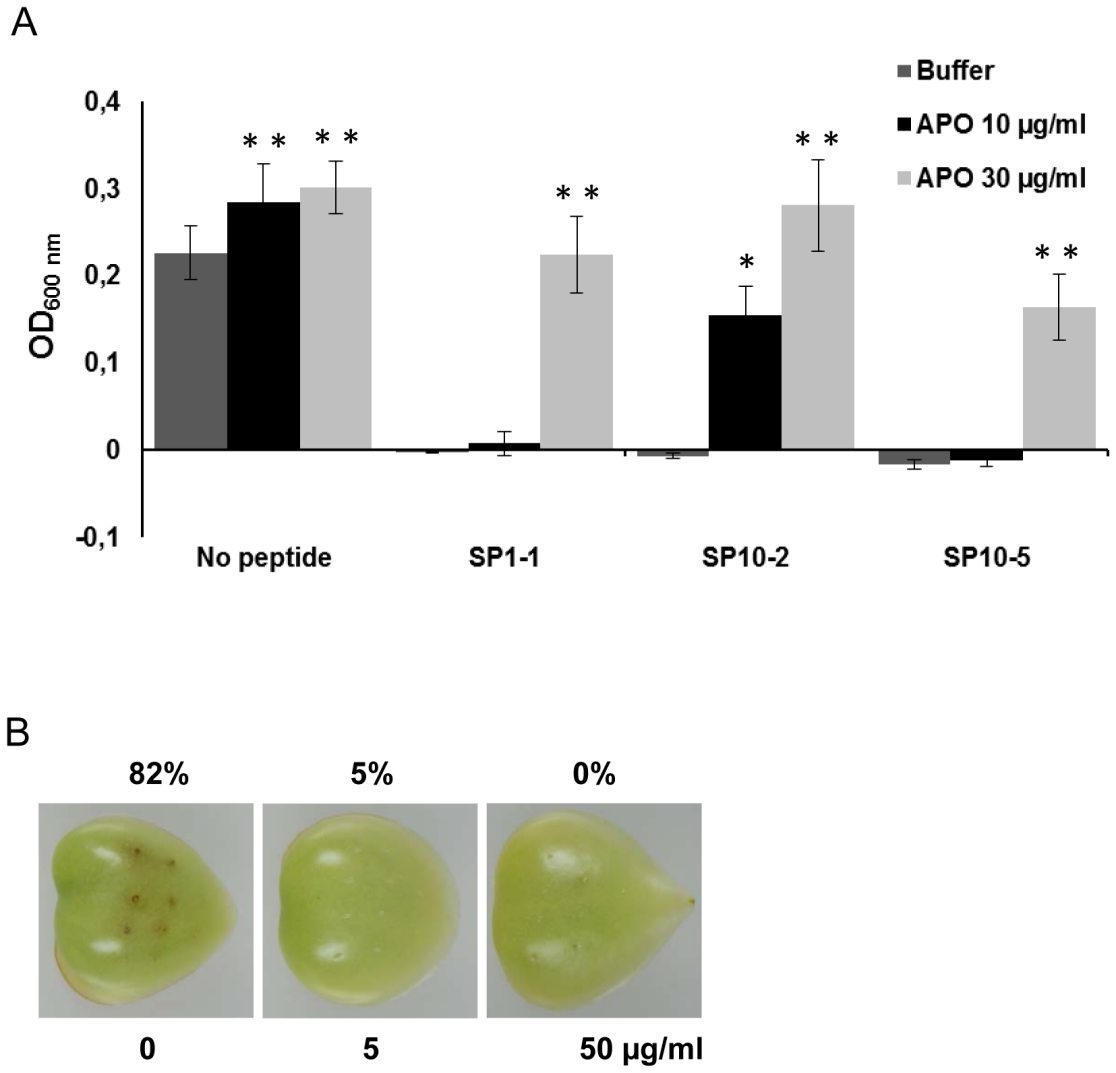
Antibacterial activity of synthetic peptides in presence of apoplast fluid and in tomato fruits. (A) Approximately 10^5^ cfu/ml bacteria (*P. syringae* pv *tomato*) were incubated with 0 or 10 µg/ml peptide in the presence or absence of different concentrations (10 µg/ml or 30 µg/ml) of tomato apoplastic fluid. After 14–16 h the bacterial growth was determined by measuring OD_600 nm_. APO, tomato apoplastic fluid. Values represent the mean of at least three biological replicates ± standard error of the mean. *indicates significantly different in comparison to the corresponding control treatment, *P*<0.05. **indicates significantly different in comparison to the corresponding control treatment, *P*<0.01. (B) *X. vesicatoria* (0.5×10^5^ cfu/ml) were treated with different concentrations of peptide SP10-5 and immediately injected into tomato fruits. After incubation for 5 d at room temperature infection symptoms were monitored. Above the values of incidence of infection symptoms is given in percentage. The total number of inoculation sides of three biological replicates were 22.

Bacteria enter plants mainly through stomata or lesions. Especially at lesions apoplast and cytoplasmic fluids are released, which may inactivate peptides sprayed onto the surface, e. g. due to protease activities. Therefore, we tested the activity of the peptides SP1-1, SP10-2 and SP10-5 against *P. syringae pv. tomato* in combination with apoplast fluid (Figure 6A). Apoplast fluid of tomato leaves inhibited the activity of all three peptides at a concentration of 30 µg/ml and the activity of SP10-2 was reduced to 50% even at concentrations of 10 µg/ml.

To evaluate the potency of our peptides in inhibiting disease progression a fruit-infection assay with *X. vesicatoria*, the causal agent of bacterial spot disease, was performed. SP10-5 is quite stable against apolastic fluids and was therefore used for fruit-infection assays. Peptide-treated *X. vesicatoria* bacteria were directly injected into tomato fruits (Figure 6B). After 5 days clear bacterial spots could be detected in fruits inoculated with peptide solvent-treated bacteria, whereas treatment with 5 or 50 µg/ml SP10-5 reduced the symptoms dramatically concluding that the peptide is also effective in planta.

## Discussion

Bacteria, fungi and viruses can dramatically affect yield and quality of crop plants, which can have enormous economic consequences. But the damage is not only due to loss of money. Moreover, some of these phytopathogenic microorganisms can produce toxins, which can affect health of the consumers. For instance aflatoxins, contaminants produced by the fungi *Aspergillus flavus* and *Aspergillus parasiticus* in a variety of food crops, are known to cause human liver cancer, to affect growth of human and animals and to be immunosuppressive [40]–[43]. Therefore, the control of the plant pathogens is not only important from the economic point of view, but also very important for human and animal health. The control of the pathogenic microorganisms relies mainly on chemical pesticides [9]. However, many countries have undertaken regulatory changes in pesticide registration requirements with the aim of retaining only compounds being more selective, with lower toxicity and reduced negative environmental impact. The use of AMPs in agriculture was already proposed along with their discovery in the early 1980s. To date, nearly thousand natural antimicrobial peptides have been isolated from different organisms, such as mammals, insects, amphibians, and plants and can be found in AMP databases [28]. The use of AMPs in animal or human medicine and plant protection may offer new possibilities to control microbial diseases that are still challenging to combat. However, most natural occurring AMPs exhibit a narrow activity spectrum, low activity against important pathogens or high toxicity against human and plant cells [44], [45]. To overcome these problems different types of antimicrobial peptides were designed [45]. We developed helical antimicrobial peptides harbouring positively charged and hydrophobic clusters. In a first approach 16 different peptides with a length of 12 and 20 amino acids were developed. All designed peptides have a positive net charge of at least 3.76 at physiological pH, as it is the case for most of the natural occurring AMPs. SP1 and SP2 differ mainly in their N-terminal half and showed a broad antibacterial activity in a concentration range of 1–2 µg/ml (MIC). Interestingly, SP3 and SP4 displayed a significant lower antibacterial activity, although the arrangement of hydrophobic and charged amino acid residues is the same as in SP1 and SP2. Notably, SP1 contains no alanine, whereas SP2, SP3 and SP4 contain two, three and four alanine residues, respectively. Wang et al. (2009) analyzed the amino acid composition of AMPs from different kingdoms of life listed in “The Antimicrobial Peptide Database (http://aps.unmc.edu/AP/main.php. Accessed 2013 July 12) and determined frequently occurring residues. The most abundant residues in bacterial peptides are glycine and alanine (>11%). Possibly, with increasing alanine content the designed peptides reflect more analogy to bacterial peptides resulting in lower antimicrobial activity. Another difference among the peptides of group I is the slightly lower hydrophobicity of SP3 and SP4. This might affect the possibility of SP3 and SP4 to interact with bacterial membranes or to adopt membrane-induced amphipathicity at the expense of inhibiting bacterial growth [4], [45].

The group II peptides with a positively charged C-terminus and a hydrophobic N-terminus and group III peptides harboring a hydrophobic and positively charged side showed quite low activity against the tested plant bacteria. Compared to SP1, which exhibited actually the highest and broadest activity against the tested plant pathogenic bacteria, the peptides from group II and III lack the positively charged N-terminus. This feature might favour the “peptide-pathogen” interaction resulting in an inhibition of bacterial multiplication. The findings of Alvarez-Bravo and coworkers [46] support this conclusion. Their experiments are based on synthetic peptides reflecting the antimicrobial active core of sapecin B, an insect AMP found in the larval hemolymph of the flesh fly (*Sarcophaga peregrina*). They generated several short peptides mainly composed of a stretch of leucine residues forming the hydrophobic core bordered by lysine and arginine containing sequences at the termini. For their activity a terminal KLK or RLK motif was critical. In addition, the length of the hydrophobic core was important for the antibacterial activity but, surprisingly, not for the antifungal properties of the peptides [46]. Hence, beside the positive net-charge also terminal structures of the peptides might contribute to the extent of their antimicrobial properties.

Within the peptides of group IV only SP13 and SP16 were active against the tested bacterial microorganisms. Especially SP13 showed similar activity against *C. michiganensis*, *X. vesicatoria*, and *P. corrugata* as SP1 and SP2 with a MIC of 2.5 µg/ml. Interestingly, in SP13 as well as in SP16 the charged terminal parts are connected via three charged amino acids. Probably this structural characteristic allows a defined pore-like incorporation into the bacterial membrane.

Interestingly, the peptides containing D-amino acids showed significant lower low hemolytic activity than the corresponding L-forms (see SP10-D and SP13-D). This was also observed in previous studies for other peptides [47]–[49]. Furthermore, these peptides are more active against fungal pathogens suggesting an increased resistance against degradation [47].

Most of the previous work on AMP is based on *in-vitro* data demonstrating the activity of the peptides in growth media. However, *in-vitro* and in*-vivo* conditions differ greatly and results obtained from *in-vitro* inhibition assays may just serve as indication for the potency of compounds in*-vivo* [45]. In the plant microenvironment complex factors might affect the interaction between peptides and pathogens. Therefore, inhibition studies on susceptible plant tissue have to be carried out to analyse the potential use of the peptides for plant protection. Most assays are based on detached leaves or leaf disks, flowers or fruits (*ex-vivo*) [12], [50]–[55]. With such assays it is possible to investigate the activity of the peptides on the plant surface, but the way they are performed did not reflect a potential way of practical application. In nature bacteria are spread by wind and rain, penetrate leaves and fruits through stomata and wounds and multiply intercellular to induce lesions on stems, leaves and especially fruits [9]. To simulate the natural inoculation, bacteria were sprayed onto the tomato leaf surface. Using this spraying technique, we could demonstrate that peptides SP1-1, SP10-2 and SP10-5 successfully inhibited the proliferation of *P. syringae pv. tomato* on tomato leaves. The higher peptide concentrations needed in the spraying assays is probably due to the higher amount of bacteria used to infect plants. Furthermore, degradation on the plant surface cannot be excluded. Especially after wounding the antimicrobial activities of the peptides are reduced. At such areas apoplast and/or tissue fluids are leaking and in such fluids the AMPs may be inactivated by protease-based degradation or by binding or reacting with phenolic compounds. Interestingly, the peptides SP1-1 and SP10-5 are significantly more resistant against apoplast fluid dependent inactivation than SP10-2. Moreover, SP10-5 is inhibiting symptom development after injection into tomato fruits. These results make peptide SP10-5 a promising candidate for transgenic approaches.

In general, the spraying technique displays a promising method for the use of antimicrobial peptides in crop plant protection and can be of special importance to fight *Erwinia amylovora*. This gram negative, facultative anaerobic bacterium causes fire blight, a destructive bacterial disease of apples and pears that kills blossoms, shoots, limbs, and sometimes entire trees [56], [57]. After infection the bacteria can spread throughout the whole tree and since there is no cure for fire blight after infection tissues must be removed by pruning. To suppress epiphytic growth and to avoid the infection process farmers applying antibiotics during bloom [58]. Until now there are just a few reports about antimicrobial peptides active against *E. amylovora* [50], [51], [59]. Currently, streptomycin sprays are used to protect plants from this deathly disease, which are most effective if applied on the day before or the day of an infection event. Since our designed peptides are active against a broad range of bacteria we cannot exclude that also beneficial bacteria on the plant surface are affected. However, growth-promoting bacteria and systemic resistance inducing bacteria are mainly associated with the plant rhizosphere [60], [61] and therefore are a kind of “protected” from the sprayed peptides.

The growth inhibiting ability on the plant surface was also shown for the synthetic peptides pep11 and pep20 [53], albeit the described assay differs from the assay system presented here. These peptides inhibited bacterial soft rot on potato tubers to 100% when the inoculum was mixed with the peptides before treatment. Additionally, the growth of *Alternaria solani* on potato leaves was markedly reduced. However, in both cases the restricted infection could be a result of direct microbe killing in the reaction tube. Next to the application onto the plant surface, production of various AMPs in transgenic plants led to protection against different plant pathogens [62]–[68].

Elucidation of the mode of action of the AMPs is important for their application and further development. Many natural antimicrobial peptides are known to act via perturbation of bacterial membranes [69]–[71]. Therefore, the membrane disturbing ability of some highly active representatives of the designed peptides was investigated. The peptides SP10-5 and 13-2 revealed strong membrane depolarization levels in range of the well described pore-forming indolicidin [72], [73]. Interestingly, SP1-1 showed only a moderate membrane depolarizing activity. Since all tested peptides have a similar MIC value (Table 4), but show different membrane depolarizing activity, we conclude that they act via different modes of action. Peptides with activities on microbial membranes or other generalized targets are often preferred to antibiotics with very distinct targets, because of lower risk of resistance development [74]. Therefore, especially SP10-5 represents a promising candidate for further development since it display high antimicrobial activity, low toxicity and a membrane based mode of action.

To point out the relevance of our results the features of the most relevant peptides identified (SP1-1, SP10-2 and SP10-5) are summarized in supplement (Table S5) and compared to the well-characterized natural peptides magainin II and protegrin I [75]–[79]. However, such a comprehensive set of data for plant protection is not available for natural peptides. Furthermore, different experimental setups – especially for *ex/in-vivo* experiments – make a comparison also difficult. The amino acid composition of our designed peptides is reduced to hydrophobic and positively charged amino acids, whereas the natural peptides are also composed of neutral and negatively charged amino acids. Furthermore, the amino acid sequence of our designed peptides is shorter than that of the natural ones. This could be of importance, in the case of that the peptide have to be produced in a synthetic way. Moreover, the growth inhibiting activity of our designed peptides is significantly higher than that of the natural ones, whereas their hemolytic activity is lower.

Taken together, we present here *de-novo* designed peptides and demonstrated their high antimicrobial activity and low host cell cytotoxicity. Their antimicrobial spectrum covered a wide range of different plant pathogenic bacteria and fungi. With the increasing demand for antimicrobial compounds for production of “healthy” food, these peptides might serve as templates for novel antimicrobial agents. The plant protecting ability of the designed peptides was demonstrated by spraying them on the plant surface. Besides the generation of transgenic plants this way of application displays a promising method for practical use of antimicrobial peptides in plant protection.

## Materials and Methods

### Peptides

Magainin II, Indolicidin, Histatin 5, and Cathepsin G (77–83) were purchased from Bachem (Weil am Rhein, Germany). Protegrin I (>95% purity) and all designed peptides (>80% purity) were synthesized from metabion (Munich, Germany). Peptides were dissolved in dilution buffer 0.01% acetic acid, 0.2% bovine serum albumin (Sigma, Munich, Germany) at a concentration of 1 mg/ml, filter sterilized and stored at −20°C.

### Microorganisms

Phytopathogenic bacteria: Pseudomonas syringae pv. syringae (DSM 10604), Pseudomonas syringae pv. tomato (DSM 50315), Pseudomonas corrugata (DSM 7228), Pectobacterium carotovorum ssp. carotovorum (DSM 30168), Clavibacter michiganensis ssp. michiganensis (DSM 46294), Xanthomonas vesicatoria (DSM 50861) and virulent Pseudomonas syringae pv. tomato DC3000 (kan^+^ rif^+^). Bacteria were stored in 20% glycerol at −80°C.

Phytopathogenic fungi: *Alternaria alternata*, *Botrytis cinerea*, *Cladosporium herbarum*, and *Fusarium solani*. Fungal cultures were grown on 2% malt extract agar at room temperature in the dark and transferred to light to induce sporulation.

### CD Spectra

Far-UV (190–260 nm) CD spectra were recorded on 130 µM samples of antimicrobial peptides (SP1-1, SP8, SP10-1 and SP13) in H_2_O and in 1 mM 1,2-Dimyristoyl-sn-glycero-3-phospho-sn-glycerol (DMPG), respectively, using a JASCO J-715 spectropolarimeter. The sample temperature for all CD measurements was maintained at 293 K. Spectra were corrected by subtraction of background (buffer and DMPG solution, respectively). Each experiment was repeated independently.

### Bacterial and Fungal *in-vitro* Assays

*In-vitro* inhibition assays were performed in sterile flat-bottom 96-well polypropylene-plates (Greiner bio-one, Frickenhausen, Germany). 10 µl (antibacterial assay) or 20 µl (antifungal assay) of each concentration were loaded per well. Fresh bacterial colonies were transferred into LB-medium, grown to an OD_600 nm_ of 0.08–0.1 and diluted 1∶100 with medium. 90 µl of this suspension containing about 10^5^ CFU/ml of bacteria were added to each well resulting in final peptide concentrations from 0 to 100 µg/ml. After incubation at 27°C for 1–2d bacterial growth was analyzed by measuring the OD_590 nm_ using a Tecan Genios microplate reader (Crailsheim, Germany).

Spores of plant pathogenic fungi were collected by washing sporulating cultures with water. Spore concentration was determined by haematocytometer and adjusted to 1.5–2×10^3^ spores/ml in 2% malt extract medium. 80 µl of fungal spores were added per well resulting in final peptide concentrations from 0 to 100 µg/ml. After incubation at room temperature for 3d on a rotary shaker (300 rpm), fungal growth was determined by measuring the OD_590 nm_ (*Alternaria sp.*) or by visual screening of the plates (*Botrytis sp.*, *Cladosporium sp.*). The lowest peptide concentration, where no visible microbial growth could be detected, was determined for each organism-peptide combination and referred to as the minimal inhibitory concentration (MIC). At least two independent replicates were performed.

### Determination of the Hemolytic Activity and Phytotoxicity

The hemolytic activity of the peptides was evaluated by determining hemoglobin release of suspensions of fresh human erythrocytes at 405 nm. Human red blood cells (Deutscher Blutspendedienst, München, Germany) were centrifuged and washed three times with Tris-buffer (10 mM Tris, 150 mM NaCl, pH 7.4). 80 µl of cells (1.5×10^9^ cells/ml) were added to 20 µl of the peptide solution in 96-well plates and incubated for 45 min at 37°C. Final peptide concentrations were 0, 20, 50, 100 and 200 µg/ml. After centrifugation (1,500 g, 5 min, 20°C) 30 µl aliquots of the supernatant were transferred to 96-well plates containing 100 µl of water and released hemoglobin was determined using a microplate reader (OD_405 nm_). 100% hemolysis was determined with 2% SDS.

Phytotoxicity was measured on Arabidopsis mesophyll protoplasts prepared from leaves as described in [80]. 80 µl protoplast suspension containing about 5000 protoplasts were incubated with 20 µl peptide solution. Final peptide concentrations were 0, 10, 100 and 200 µg/ml. The phytotoxic effect of antimicrobial peptides was observed after 1 h with a light microscope. Hints to cell death are loss of spherical shape, chloroplast release and/or agglomeration of protoplasts.

### Bacterial Membrane Potential Measurements

The peptidés potential to depolarize bacterial membranes was determined by a method of Friedrich et al. [36], [72]. Bacteria (*P. syringae pv. syringae* or *C. michiganensis pv. michiganensis*) were grown overnight, diluted to a OD_600 nm_ of 0.01 and grown at 30°C to the mid-logarithmic phase (OD_600 nm_: 0.4–0.5). Cells were harvested by centrifugation (3,000 g, 4°C, 10 min). They were washed twice with 5 mM HEPES, pH 7.2 and diluted to a OD_600 nm_ of 0.05 (5×10^7 ^cfu/ml) in the same buffer with addition of 0.1 M KCl and incubated for 10 min at 30°C to equilibrate the outer and inner side of the membrane. Then the membrane potential sensitive dye DiSC_3_(5) (3,3′-dipropylthiadicarbocyanine iodide) was added to a final concentration of 0.4 µM and the cells were incubated for further 30 min until the reduction in fluorescence was stable (about 90%). Peptides in final concentrations of 0.5, 1, 5, or 10 µg/ml in dilution buffer (0.01% acetic acid, 0.2% bovine serum albumin) were added and changes in membrane potential were recorded by detection of the dye (λ_ex_: 622 nm, λ_em_: 670 nm) 60 min after peptide addition with a Tecan Safire 2 microplate reader (Tecan, Crailsheim, Germany). 1% SDS and SP8 were used as positive and negative controls, respectively.

### Determination of Antimicrobial Peptide Activity on Plant Surface

Tomato plants (Microtom) were inoculated by spraying of 200 µl (per leaf) suspension of virulent *P. syringae pv. tomato* DC3000 (OD_600 nm_ of 0.01, approximately 10^7^ CFU/ml; kan^+^, rif^+^) from a distance of about 20 cm using an aerosol can. In this way approximately 50 µl bacteria suspension was applied directly onto the leaf surface. After 30 min different concentrations of the peptides were sprayed onto the inoculated leaves (two leaves for each concentration) and kept at room temperature for 30 min. Two leaf disks per leaf were punched out using a 1.5 ml Eppendorf tube, transferred into 500 µl HPG-medium (0.5% yeast extract, 1% peptone, 0.1% glucose) and vortexed intensively to resuspend the bacteria. 200 µl were plated onto LB-agar plates containing 50 µg/ml kanamycin and 50 µg/ml rifampicin and colonies were counted after incubation for two days at 28°C.

To determine antifungal activity of AMPs four week old tomato plants (Microtom) were inoculated with fungal spores using an aerosol can. Spores were harvested by washing sporulating plate-grown fungi (*A. alternata* and *C. herbarum*) with 62.5 mM KH_2_PO_4_ (pH 6.0) supplemented with 5.5 mM glucose and 0.1% Tween-20. One leaf per plant (three plants per treatment) was sprayed adaxial and abaxial with 500 µl spore suspension (10^4^ spores/ml). In this way approximately 100 µl spore suspension was applied directly onto the plant surface. Plants were covered with a plastic cap to guarantee high humidity and incubated at 20°C. After two hours the T_0_ tissue sample was collected. AMPs were sprayed onto the leaf surface 22 h after inoculation. Leaves were harvested 24 h and 48 h after peptide treatment. Frozen material was homogenized in liquid nitrogen and fungal DNA was isolated [81]. Quantification of fungal DNA content was done by qRT-PCR amplifying the nuclear encoded ribosomal internal transcribed spacer (ITS) regions [82]. ITS1-forward and ITS4-reverse primers were used at an annealing temperature of 60°C: For *A. alternata*: ITS1-Alt*-*for, 5′-TCTAGCTTTGCTGGAGACTC-3′ and ITS4_Alt-rev, 5′-AGACCTTTGCTGATAGAGAAGT-3′ and for *C. herbarum*: ITS1-Cla-for 5′-CAAACTCTTGCGTAACTTTGC-3′ and ITS4-Cla-rev, 5′-CACAACGCTTAGGGGACAG-3′ (synthesized by metabion, Munich, Germany). All spraying experiments were done in at least three biological replicates. Differences between the mean values were analysed using an unpaired t-test analysis of variance.

### Extraction of Apoplastic Fluid and Determination of Antibacterial Activity of Synthetic Peptides in Presence of Apoplast Fluid

Apoplastic fluid was extracted using vacuum infiltration [83]. Therefore, tomato leaves were cut from 6–8 weeks old plants washed with distilled water and dried using tissue paper. Eight to ten leaves were placed in a 50-ml recipe containing 30 ml of sterile deionized water. Cycles of pressure and vacuum were applied carefully until leaves were completely infiltrated. The infiltrated leaves were dried with tissue paper, rolled and put into a 5 ml syringe (without cannula). The syringe was placed in a 15-ml conical falcon tube. The apoplast extract was collected by spinning the conical tubes at 2,.225 g for 20 min at 4°C. Protein concentration of the apoplastic fluid was determined using the Bradford assay.

Approximately 10^5^ cfu/ml bacteria (*P. syringae* pv *tomato,* OD_600 nm_ of 10^−4^) were incubated with 0 or 10 µg/ml peptide in the presence or absence of different concentrations (10 µg/ml or 30 µg/ml) of tomato apoplastic fluid. After 14–16 h bacterial growth was determined by measuring OD_600 nm_. Experiments were done in at least three biological replicates. Differences between the mean values were analysed using an unpaired t-test analysis of variance.

### Inoculation of Tomato Fruits

Immature unripe fruits were detached with cotton wool soaked in 80% ethanol, cut into four pieces and placed on ½ MS-Agar plates. Bacterial cells (*X. vesicatoria*, OD_600 nm_ of 0.002, approximately 0.5×10^5^ CFU/ml) were mixed with peptide solvent (0.01% acetic acid, 0.2% bovine serum albumin) or different concentrations of peptides (0, 5 or 50 µg/ml) and inoculated immediately by injecting 1 µl in tomato fruits. Dependent on the size of the tomatoes a fruit was inoculated at up to ten positions. The fruits were kept in a humid chamber and incubated at room temperature. The infection process was monitored daily and after 3–5 days infection sides were counted. The experiment was done in three biological replicates. Per replicate one fruit for each peptide concentration has been used.

## Supporting Information

Figure S1 **Strategy for peptide design.**
(PDF)

Figure S2 **NMR-based structural analysis of SP1-1.**
(PDF)

Figure S3 **Effect of SP15, SP7-D and SP10-D on the viability of Arabidopsis mesophyll protoplasts** ***in vitro*** **.**
(PDF)

Figure S4 **Time course of fungal growth on tomato leaves treated with different peptide concentration.**
(PDF)

Table S1 **Antimicrobial activities (MIC) of naturally occurring peptides.**
(PDF)

Table S2 **Sequences and structural-chemical properties of D-amino acid modified peptides.**
(PDF)

Table S3 **Antimicrobial activities (MIC) of D-amino acid modified peptides against plant pathogens and their hemolytic activities.**
(PDF)

Table S4 **Sequences and structural-chemical properties of peptides of the 2^nd^ generation.**
(PDF)

Table S5 **Features of SP1-1, SP10-2 and SP10-5 in comparison to the natural peptides magainin II and protegrin I.**
(PDF)
