# Supplementary material for: De-Novo Design of Antimicrobial Peptides for Plant Protection
Source: PLoS One. 2013 Aug 12;8(8):e71687. doi: 10.1371/journal.pone.0071687 (PMC3741113; doi:10.1371/journal.pone.0071687)
Supplement: Figure S1 — Strategy for peptide design. (PDF) [file pone.0071687.s001.pdf]

Step 1: **Template selection**

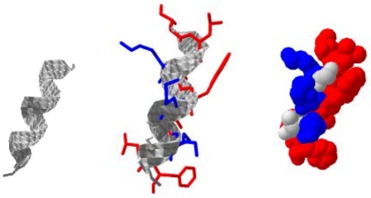

IsCT derivative
PDB code: 1T52

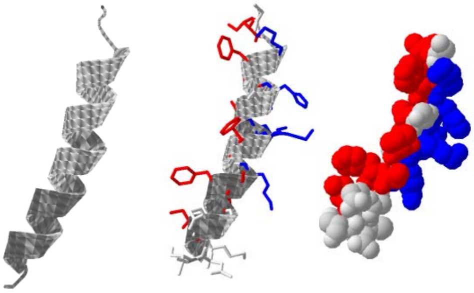

Magainin II
PDB code: 2MAG

Step 2: **First generation**

IsCT: ILGKIWKGIKSLF

e.g. SP1: RKKRLKLLKRLV-

Magainin II: GIGKFLHSAKKFGKAFVGEIMNS

e.g. SP13: -KRRLIARILRLAARALVKKR--

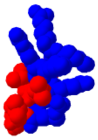

SP1
group I

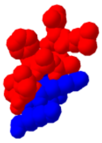

SP5
group II

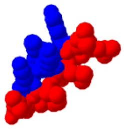

SP9
group III

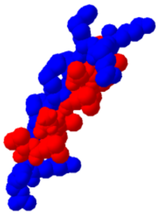

SP13
group IV

Step 3: **Second generation**

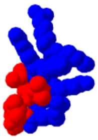

SP1
group I

SP1: RKKRLKLLKRLV

SP1-1: RKKRLKLLKRLL

Figure S1. Strategy for peptide design.

Step 1: Selecting templates for helical structure from the Protein Data Bank (PDB website; http://www.rcsb.org/pdb/home/home.do. Accessed 2013 July 12). We selected a derivative of the scorpion-derived antimicrobial peptide IsCT (for 12 AA peptides) and the frog-derived peptide magainin II (for 20 AA peptides) as templates.

Step 2: Using the mutation tool of the SWISS-pdbviewer software four groups of peptides were designed differing in location and size of charged and hydrophobic clusters. A positive net charge of the designed peptides was guaranteed by using arginine, lysine, and histidine residues in the sequence. Leucine, isoleucine, valine, phenylalanine, alanine, methionine, glycine, serine, and threonine residues were used to generate hydrophobic regions. A helical structure of the peptides was ensured by inserting strong helix-forming amino acids, such as leucine and alanine. The software enables to see directly a structural model of the designed peptides.

In this way four leading structures (group I – IV) have been designed. Peptides of group I consist of a dominant charged cluster and a small hydrophobic region. Group II contains peptides with a dominant hydrophobic cluster and a small charged region. In all peptides of group III the hydrophobic and the charged regions have the same size and are separated lengthwise of the molecule. In peptides of group IV the charged regions are located at the N- and C-termini, which are separated by a central hydrophobic cluster. In peptides SP13 and SP16 the charged N-terminal and C-terminal parts are connected by a charged bar. Within each group four peptides were designed by varying charge and/or hydrophobicity.

Step 3: Second generation
Peptides from three different groups have been selected as lead structures to develop a second generation of peptides by directed exchange of distinct amino acids. SP1 (group I) has been selected, since it is the most active one of all designed peptides. SP10 (group III) has been selected, since it is the only peptide active against *Pectobacterium carotovorum*. This bacterium is related to *Erwinia amylovora* - a very dangerous pathogen for plants of the subfamily Pomoideae (e. g. apple and pear trees). Finally, the best peptide of group IV (SP13) has been selected. The design of the 2nd generation aimed to get peptides with higher antimicrobial activity and lower hemolytic activity than the corresponding lead structures. Therefore, the amino acid sequence of the lead structures has been changed resulting in special features of the second generation peptides (see **Table 3**).
