# Supplementary material for: De-Novo Design of Antimicrobial Peptides for Plant Protection
Source: PLoS One. 2013 Aug 12;8(8):e71687. doi: 10.1371/journal.pone.0071687 (PMC3741113; doi:10.1371/journal.pone.0071687)
Supplement: Figure S2 — NMR-based structural analysis of SP1-1. (PDF) [file pone.0071687.s002.pdf]

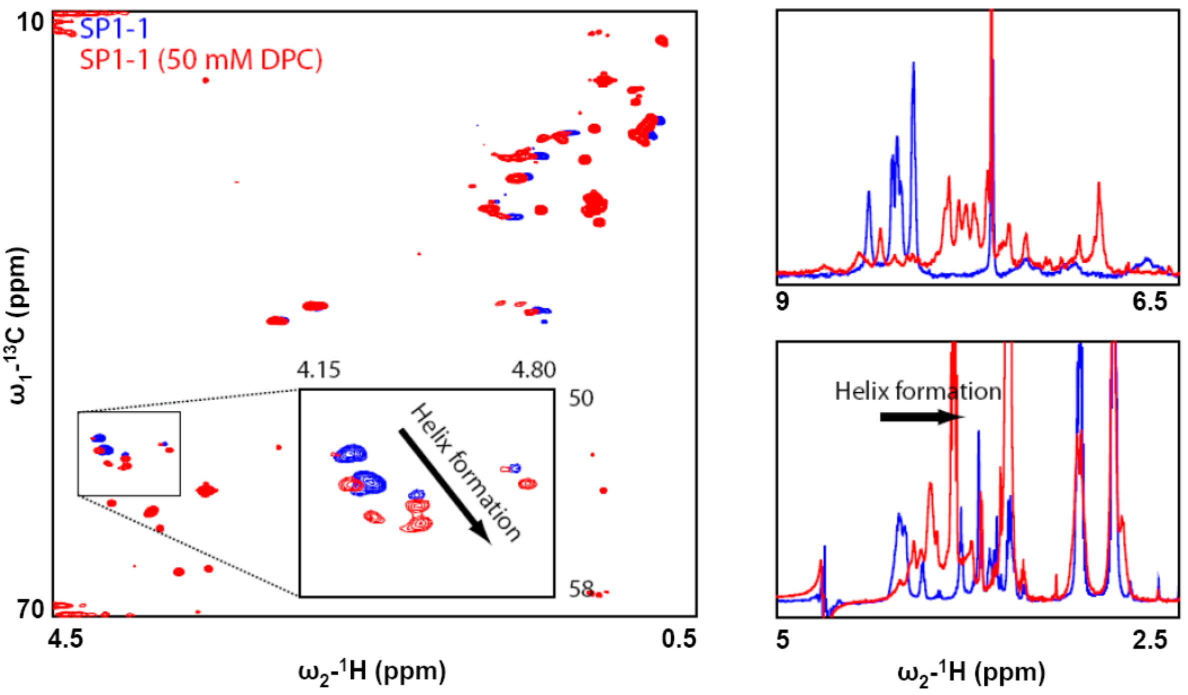

Figure S2: NMR-based structural analysis of SP1-1.
^1H, ^{13}C HSQC and ^1H NMR spectra of 1 mM SP1-1 alone (blue) and in the presence of 50 mM perdeuterated dodecylphosphocholine (DPC) (red). The chemical shift changes of H_α signals to lower and of $^{13}C_\alpha$ frequencies to higher ppm values are characteristic for the formation of an α-helical conformation.
