# Supplementary material for: De-Novo Design of Antimicrobial Peptides for Plant Protection
Source: PLoS One. 2013 Aug 12;8(8):e71687. doi: 10.1371/journal.pone.0071687 (PMC3741113; doi:10.1371/journal.pone.0071687)
Supplement: Figure S3 — Effect of SP15, SP7-D and SP10-D on the viability of Arabidopsis mesophyll protoplasts in vitro . (PDF) [file pone.0071687.s003.pdf]

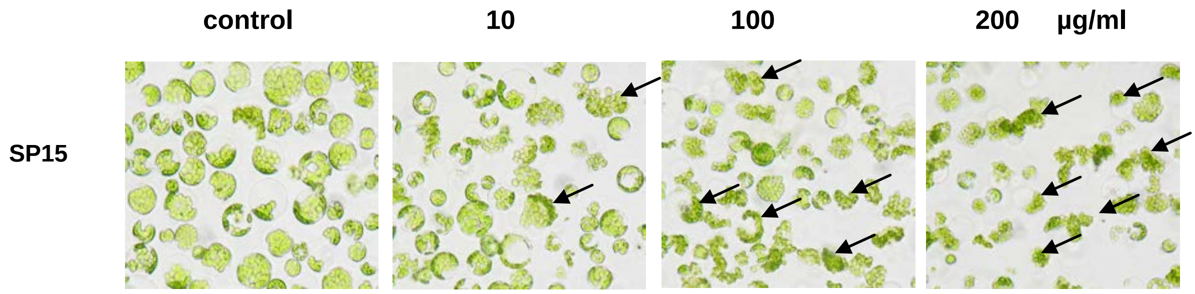

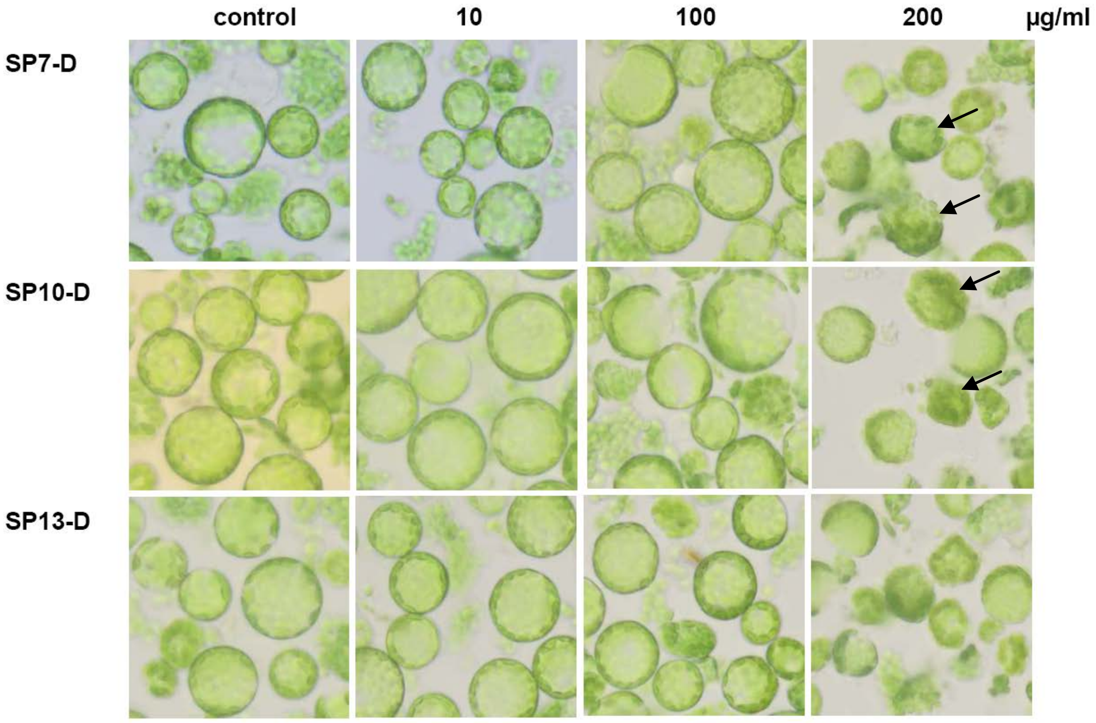

Figure S3: Effect of SP15, SP7-D and SP10-D on the viability of Arabidopsis mesophyll protoplasts *in vitro*.

Protoplasts were incubated with different peptide concentrations and photographs were taken after 1 h. Cells with spherical shape without any sign of cytoplasmic degradation were defined as viable. Treatment with SP15 - a highly phytotoxic peptide – has been used as positive control for phytotoxic effects. Hints to cell death include loss of spherical shape, chloroplast release and agglomeration of protoplasts (arrows) and can be observed with SP15 at low concentrations.
