# Supplementary material for: De-Novo Design of Antimicrobial Peptides for Plant Protection
Source: PLoS One. 2013 Aug 12;8(8):e71687. doi: 10.1371/journal.pone.0071687 (PMC3741113; doi:10.1371/journal.pone.0071687)
Supplement: Figure S4 — Time course of fungal growth on tomato leaves treated with different peptide concentration. (PDF) [file pone.0071687.s004.pdf]

Growth inhibition of *Cladosporium herbarum* by SP13-D

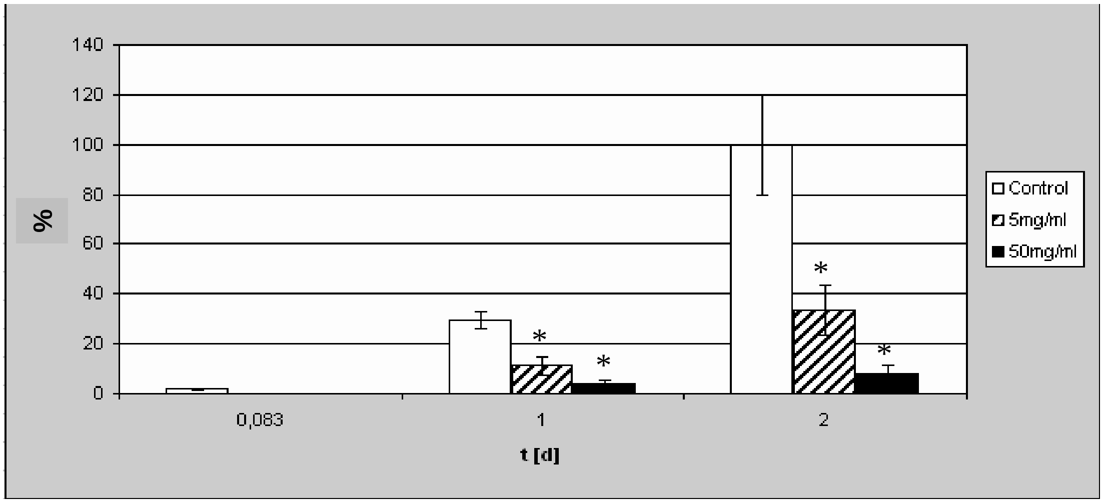

Growth inhibition of *Alternaria alternata* by SP10-D

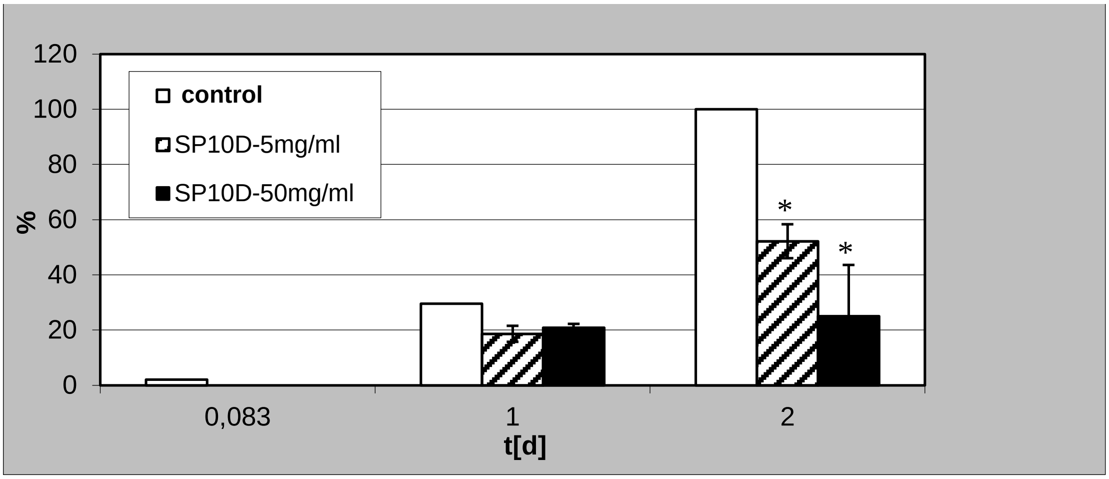

Figure S4. Time course of fungal growth on tomato leaves treated with different peptide concentration.

Tomato leaves were inoculated with spores of *Cladosporium herbarum* or *Alternaria alternata* or (10^4 spores/ml). After 22 h different concentrations of antimicrobial peptides were sprayed onto the leaves. Fungal growth was analysed at the indicated time points by quantification of fungal DNA content in the leave tissue. Fungal growth on control leaves after 2 days was set to 100%. Values represent the mean of three biological replicates ± standard error of the mean. *; indicates significantly lower than the control treatment, $P<0.05$.
