# Supplementary material for: De-Novo Design of Antimicrobial Peptides for Plant Protection
Source: PLoS One. 2013 Aug 12;8(8):e71687. doi: 10.1371/journal.pone.0071687 (PMC3741113; doi:10.1371/journal.pone.0071687)
Supplement: Table S1 — Antimicrobial activities (MIC) of naturally occurring peptides. (PDF) [file pone.0071687.s005.pdf]

Table S1. Antimicrobial activities (MIC) of naturally occurring peptides.

Organism	Protegrin I	Cathepsin G	Histatin 5	Indolicidin	Magainin II
			**µg/ml**		
**Fungi**					
Botrytis cinerea	64	> 64	64	32	64
Alternaria alternata	32	> 64	> 64	32	> 64
Fusarium solani	8	> 64	16	8	2
Cladosporium herbarum	> 64	> 64	> 64	64	64
**Bacteria**					
Clavibacter michiganensis ssp. michiganensis	< 1	> 64	> 64	< 1	32
Pectobacterium carotovorum ssp. carotovorum	> 64	> 64	> 64	> 64	> 64
Xanthomonas vesicatoria	2	> 64	> 64	2	8
Pseudomonas syringae pv. tomato	4	> 64	> 64	> 64	32
Pseudomonas syringae pv. syringae	32	> 64	> 64	> 64	> 64
Pseudomonas corrugata	8	> 64	> 64	> 64	32

Several AMPs from human or animal origin are commercially available, such as cathepsin G and histatin 5 (human), protegrin I (pig), indolicidin (bovine) and magainin II (frog). The peptides were incubated with the different microorganisms at a concentration range from 1 to 64 µg/ml. Cathepsin G and histatin 5 showed nearly no growth inhibition – even not at the highest concentrations used in the test assay. Only the growth of *Fusarium solani* is inhibited by histatin 5 at 16 µg/ml. Only protegrin I, indolicidin and magainin II showed activities in the single-digit range against some plant pathogens.

Histatin-5
DSHAKRHHGTKRKFHEKHHSHRGT (BACHEM, Weil am Rhein, Germany)

Cathepsin G (77-83)
HPQTNQR (BACHEM, Weil am Rhein, Germany)

Magainin II
GIGKFLHSAKKFGKAFVGEIMNS (BACHEM, Weil am Rhein, Germany)

Indolicidin
ILPWKWPWWPWRR (BACHEM, Weil am Rhein, Germany)

Protegrin 1
RGGRLCYCRRRFCVCVGR (METABION, München, Germany)
