# Supplementary material for: De-Novo Design of Antimicrobial Peptides for Plant Protection
Source: PLoS One. 2013 Aug 12;8(8):e71687. doi: 10.1371/journal.pone.0071687 (PMC3741113; doi:10.1371/journal.pone.0071687)
Supplement: Table S2 — Sequences and structural-chemical properties of D-amino acid modified peptides. (PDF) [file pone.0071687.s006.pdf]

Table S2. Sequences and structural-chemical properties of D-amino acid modified peptides.

Peptide	Amino acid sequence	Charge at pH	pI[b]	H [peptide][b]	H [cluster][c]	Secondary structure
SP1-D	RK*K*RL*K*L*LK*RLV-NH_2	+ 6.76	12.31	- 0.808	2.66	---HHHHHHHH-
SP7-D	LL*I*KF*L*K*R*F*I*KH-NH_2	+ 3.84	11.26	0.550	4.27	-HHHHHHHHH--
SP10-D	L*R*FL*K*KI*L*KH*L*F-NH_2	+ 3.84	11.26	0.492	4.07	-HHHHHHHHH--
SP13-D	KR*R*LIA*R*I*L*RLAARA*L*V*K*KR-NH_2	+ 8.76	12.70	- 0.155	5.12	---HHHHHHHHHHHHHH---

[a]Estimated using the program Vector NTI 9.1 (Invitrogen). [b]Calculated using ProtParam tool (http://www.expasy.org/tools/protparam.html, [1]), H [peptide], grand average hydrophobicity of full peptide. [c]H [cluster], hydrophobicity of the hydrophobic cluster of the peptides with the calculation based on the hydrophobicity scales for amino acids [2]. [d]Secondary structure prediction according to NNPREDICT; H, helix; E, strand; -, no prediction [3]. pI, isoelectric point; D-amino acids in italics.

References

1. Gasteiger E, Hoogland C, Gattiker A, Duvaud S, Wilkins MR, et al. (2005) Protein Identification and Analysis Tools on the ExPASy Server. (In) John M Walker (ed): The Proteomics Protocols Handbook, Humana Press pp. 571-607
2. Eisenberg D (1984) Three-Dimensional Structure of Membrane and Surface Proteins. Annual Review of Biochemistry 53: 595-623.
3. Kneller DG, Cohen FE, Langridge R (1990) Improvements in protein secondary structure prediction by an enhanced neural network. J Mol Biol 214: 171-182.
