# Supplementary material for: De-Novo Design of Antimicrobial Peptides for Plant Protection
Source: PLoS One. 2013 Aug 12;8(8):e71687. doi: 10.1371/journal.pone.0071687 (PMC3741113; doi:10.1371/journal.pone.0071687)
Supplement: Table S3 — Antimicrobial activities (MIC) of D-amino acid modified peptides against plant pathogens and their hemolytic activities. (PDF) [file pone.0071687.s007.pdf]

Table S3. Antimicrobial activities (MIC) of D-amino acid modified peptides against plant pathogens and their hemolytic activities.

Organism	*SP1-D*	*SP7-D*	*SP10-D*	*SP13-D*
	**µg/ml**			
**Fungi**				
Botrytis cinerea	> 40	20	10	> 40
Alternaria alternata	20	5	5	20
Cladosporium herbarum	20	5	5	5
**Bacteria**				
Clavibacter michiganensis ssp. michiganensis	40	5	5	10
Pectobacterium carotovorum ssp. carotovorum	> 40	40	> 40	> 40
Xanthomonas vesicatoria	40	10	2	2
Pseudomonas syringae pv. tomato	5	2.5	0.5	1
Pseudomonas syringae pv. syringae	5	2.5	2.5	2.5
Pseudomonas corrugata	20	5	2.5	40
Hemolytic activity*	> 200	> 200	> 200	> 200

*Shown are the peptide concentrations leading to 25% hemoglobin release from human blood cells. > 200 describes a slight hemolytic activity at 200 µg/ml but still below the above mentioned threshold.
