# Supplementary material for: De-Novo Design of Antimicrobial Peptides for Plant Protection
Source: PLoS One. 2013 Aug 12;8(8):e71687. doi: 10.1371/journal.pone.0071687 (PMC3741113; doi:10.1371/journal.pone.0071687)
Supplement: Table S4 — Sequences and structural-chemical properties of peptides of the 2nd generation. (PDF) [file pone.0071687.s008.pdf]

Table S4. Sequences and structural-chemical properties of peptides of the 2nd generation.

Peptide	AA sequence	Charge at pH 7[a]	pI[b]	H [peptide][b]	H [cluster][c]	Special features	Secondary structure prediction[d]
**SP1**	RKKRLKLLKRLV-NH_2	+ 6.76	12.31	- 0.808	2.66		---HHHHHHHH-
**SP1-1**	RKKRLKLLKRLL-NH_2	+ 6.76	12.31	-0.842	2.65	> L	---HHHHHHHH-
**SP1-2**	RKKRVKLLKRLV-NH_2	+ 6.76	12.04	-0.725	2.67	> V	----HHHHHHH-
**SP1-3**	RKKKVKLLKRLV-NH_2	+ 6.76	12.04	-0.725	2.67	> VK	----HHHHHHH-
**SP1-4**	RKKRLKVVKRLV-NH_2	+ 6.76	12.31	-0.742	2.68	> V	----HHHHH---
**SP1-5**	RKKRLRVVRRLV-NH_2	+ 6.76	12.60	0.842	2.68	> RV	----HHHHH---
**SP1-6**	RKKKLKVVKRLV-NH_2	+ 6.76	12.04	-0.692	2.68	> KV	----HHHHHH--
**SP1-7**	RKKKLKIIKRLI-NH_2	+ 6.76	12.04	-0.617	3.25	> hydrophob, > KI	----HHHHHH--
**SP1-8**	RKKKIKIIKRLI-NH_2	+ 6.76	12.04	-0.558	3.45	> hydrophob, > KI	----HHHHHH--
**SP1-9**	RKKKIKIIKKII-NH_2	+ 6.76	11.43	-0.450	3.65	> hydrophob, > KI	----HHHHH---
**SP1-10**	RKKKAKIIKKII-NH_2	+ 6.76	11.43	-0.675	3.17	> hydrophob, > KI	----HHHHHH--
**SP1-11**	RKKKLKFFKRLF-NH_2	+ 6.76	12.04	-1.042	2.89	> hydrophob, > KF	----HHHHHH--
**SP1-12**	RKKKFKFFKRLF-NH_2	+ 6.76	12.04	-1.125	2.97	> hydrophob, > KF	----HHHHHH--
**SP1-13**	RKKKFKFFKRFF-NH_2	+ 6.76	12.04	-1.208	3.05	> hydrophob, > KF	----HHHHH---
**SP1-14**	RKKKFKIFKRLF-NH_2	+ 6.76	12.04	-0.983	3.09	> hydrophob, > KF	----HHHHHH--
**SP1-15**	KRKKLLKRLL-NH_2	+ 5.76	12.03	-0.940	2.12	< + charge, < hydrophob, > L	---HHHHHH-
**SP1-16**	KRKKLLKRLI-NH_2	+ 5.76	12.03	-0.870	2.32	< + charge, < hydrophob, > L	---HHHHHH-
**SP1-17**	KKKKIIKRLI-NH_2	+ 5.76	11.39	-0.670	2.72	< + charge, > hydrophob, > KI	---HHHHH--
**SP1-18**	RKKRKKLLKRLL-NH_2	+ 7.76	12.32	-1.483	2.12	> + charge, < hydrophob, > L	----HHHHHHH-
**SP1-19**	RKKRKKLIKRLI-NH_2	+ 7.76	12.32	-1.367	2.52	> + charge, < hydrophob, > K	----HHHHH-H-
**SP1-20**	RKKRKKLLKRLI-NH_2	+ 7.76	12.32	-1.425	2.32	> + charge, < hydrophob, > KL	----HHHHHHH-
**SP1-21**	RKKKKKIIKKLI-NH_2	+ 7.75	11.47	-1.208	2.72	> + charge, > hydrophob, > KI	----HHHHHH--
**SP1-22**	KKKKKKIIKKII-NH_2	+ 7.75	10.85	-1.100	2.92	> + charge, > hydrophob, > KI	----HHHHHH--
**SP10**	LRFLKKILKHLF-NH_2	+ 3.84	11.26	0.492	4.07		-HHHHHHHHH--
**SP10-1**	LRFLKKILKKLF-NH_2	+ 4.76	11.33	0.433	4.07	> + charge, > K	-HHHHHHHHH--
**SP10-2**	LRFLKKALKKLF-NH_2	+ 4.76	11.33	0.208	3.59	> + charge, < hydrophob, > KA	-HHHHHHHHHH-
**SP10-3**	LRFAKKALKKLF-NH_2	+ 4.76	11.33	0.042	3.31	> + charge, < hydrophob, > KA	-HHHHHHHHHH-
**SP10-4**	LRFIKKILKKLI-NH_2	+ 4.76	11.33	0.633	4.33	> + charge, > hydrophob, > KI	-HHHHHHHHH--
**SP10-5**	LRIIKKILKKLI-NH_2	+ 4.76	11.33	0.775	4.51	> + charge, > hydrophob, > KI	-HHHHHHHHH--
**SP10-6**	LRIIRRILRRLI-NH_2	+ 4.76	12.60	0.575	4.51	> + charge, > hydrophob, > RI	--HHHHHHHH--
**SP10-7**	LRILRRLLRRLF-NH_2	+ 4.76	12.60	0.317	3.99	> + charge, > RL	-HHHHHHHHH--
**SP10-8**	LRFLRRILRRLL-NH_2	+ 4.76	12.60	0.158	3.99	> + charge, > RL	-HHHHHHHHH--
**SP10-9**	LRFARRALRRLF-NH_2	+ 4.76	12.60	-0.158	3.31	> + charge, < hydrophob, > RA	-HHHHHHHHH--
**SP10-10**	LRKLKKILKKLF-NH_2	+ 5.76	11.39	-0.125	3.46	> + charge, < hydrophob, > K	-HHHHHHHHH--

Peptide	AA sequence	Charge at pH 7[a]	pI[b]	H [peptide][b]	H [cluster][c]	Special features	Secondary structure prediction[d]
**SP10-11**	LRKAKKIAKKLF-NH_2	+ 5.76	11.39	-0.458	2.90	> + charge, < hydrophob, > KA	--HHHHHHHH--
**SP13**	KRRLIARILRLAARALVKKR-NH_2	+ 8.76	12.70	- 0.155	5.12		---HHHHHHHHHHHHHH---
**SP13-1**	KRRLIARILRLAIRALVKKR-NH_2	+ 8.76	12.70	-0.020	5.60	> hydrophob, > I	---HHHHHHHHHHHHHH---
**SP13-2**	KRRLILRILRLAIRALVKKR-NH_2	+ 8.76	12.70	0.080	5.88	> hydrophob, > IL	---HHHHHHHHHHHHHH---
**SP13-3**	KRRLILRILRLAIRILVKKR-NH_2	+ 8.76	12.70	0.215	6.36	> hydrophob, > IL	---HHHHHHHHHHHHHH---
**SP13-4**	KRRLIFRILKLFFRFLVKKR-NH_2	+ 8.76	12.61	0.075	6.56	> hydrophob, > F	---HHHHHHHHHHHHHH---
**SP13-5**	KRRILIRILKLIIKLILKKR-NH_2	+ 8.76	12.49	0.425	7.03	> hydrophob, > KIL	---HHHHHHHHHHHHHH---
**SP13-6**	KRRKLIKILKLIIKLIRKKR-NH_2	+ 10.75	12.49	-0.380	5.77	> + charge, > hydrophob, > KIL	---HHHHHHHHHHHHHH---
**SP13-7**	KRRKLIKILKLIAKLIRKKR-NH_2	+ 10.75	12.49	-0.515	5.29	> + charge, > hydrophob, > KIL	---HHHHHHHHHHHHHH---
**SP13-8**	KRRKAIKILKLIAKLIRKKR-NH_2	+ 10.75	12.49	-0.615	5.01	> + charge, < hydrophob, > KIL	----HHHHHHHHHHHHH---
**SP13-9**	KRRKAIKILKLIAKAIRKKR-NH_2	+ 10.75	12.49	-0.715	4.73	> + charge, < hydrophob, > KI	----HHHHHHHHHHHHH---
**SP13-10**	KRRLALFRAFRLALKSVLKK-NH_2	+ 7.76	12.48	-0.010	4.90	< + charge, < hydrophob	---HHHHHHHHHHHHHH---
**SP13-11**	KRRLALFRLFRLALKLVLKK-NH_2	+ 7.76	12.48	0.320	5.97	< + charge, > hydrophob	---HHHHHHHHHHHHHHH—
**SP13-12**	KRRLFLFRLFRLFLRLFLKK-NH_2	+ 7.76	12.61	0.320	6.76	< + charge, > hydrophob, > F	---HHHHHHHHHHHHHHH-
**SP13-13**	KRRKLAFRAFRFALKAVLKK-NH_2	+ 8.76	12.49	-0.315	4.96	< hydrophob, > F	----HHHHHHHHHHHHHH--
**SP13-14**	KRRKLAFRLFRLFLKLVLKK-NH_2	+ 8.76	12.49	-0.015	5.80	> hydrophob, > FL	----HHHHHHHHHHHHHH--

[a]Estimated using the program Vector NTI 9.1 (Invitrogen). [b]Calculated using ProtParam tool (http://www.expasy.org/tools/protparam.html, [1]), H [peptide], grand average hydrophobicity of full peptide. [c]H [cluster], hydrophobicity of the hydrophobic cluster of the peptides with the calculation based on the hydrophobicity scales for amino acids [2]. [d]Secondary structure prediction according to NNPREDICT; H, helix; E, strand; -, no prediction [3]. pI, isoelectric point; hydrophobic amino acids in red letters, charged amino acids in blue letters. Special features: Important alterations in comparison to the leading structure are highlighted. > increased, < decreased

References

1. Gasteiger E, Gattiker A, Hoogland C, Ivanyi I, Appel RD, et al. (2003) ExPASy: The proteomics server for in-depth protein knowledge and analysis. Nucleic Acids Res 31: 3784-3788.
2. Eisenberg D (1984) Three-dimensional structure of membrane and surface proteins. Annu Rev Biochem 53: 595-623.
3. Kneller DG, Cohen FE, Langridge R (1990) Improvements in protein secondary structure prediction by an enhanced neural network. J Mol Biol 214: 171-182.
