# Supplementary material for: De-Novo Design of Antimicrobial Peptides for Plant Protection
Source: PLoS One. 2013 Aug 12;8(8):e71687. doi: 10.1371/journal.pone.0071687 (PMC3741113; doi:10.1371/journal.pone.0071687)
Supplement: Table S5 — Features of SP1-1, SP10-2 and SP10-5 in comparison to the natural peptides magainin II and protegrin I. (PDF) [file pone.0071687.s009.pdf]

Table S5. Features of SP1-1, SP10-2 and SP10-5 in comparison to the natural peptides magainin II and protegrin I.

	SP1-1	SP10-2	SP10-5	Magainin II	Protegrin I
Amino acid sequence[a]	RKKRLKLLKRLL-NH_2	LRFLKKALKKLF-NH_2	LRIIKKILKKLI-NH_2	GIGKFLHSAKKFGKAFVGEIMNS	RGGRLCYCRRRFCVCVGR
Structural features[a]	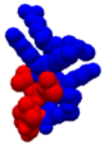	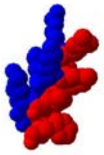	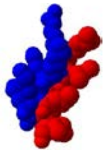	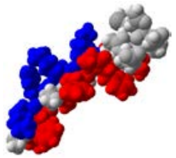	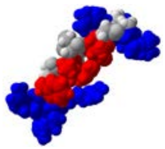
Strain specificity	*P. corrugata*<br>*C. michiganensis*<br>*P. syringae pv syrimgae*<br>*X. vesicatoria*	*P. corrugata*<br>*C. michiganensis*<br>*P. syringae pv syringae*<br>*X. vesicatoria*<br>*P. syringae pv tomato*<br>*P. carotovorum*	*P. corrugata*<br>*C. michiganensis*<br>*P. syringae pv syringae*<br>*X. vesicatoria*<br>*P. syringae pv tomato*<br>*P. carotovorum*	*P. corrugata*<br>*X. vesicatoria*<br>*P. syringae pv tomato*	*P. corrugata*<br>*C. michiganensis*<br>*X. vesicatoria*<br>*P. syringae pv tomato*
Activity range[b] [µg/ml]	0,1 – 2,5	0,1 – 1,0	0,25 – 2,5	8 – 32	1 - 8
Hemolytic activity[c] [µg/ml]	-	> 200	-	5<br>(Zasloff, 1987)	< 80<br>(Ostberg and Kaznessis, 2005)
Phytotoxicity[d] [µg/ml]	200	200	200	n.d.	n.d.
Membrane activity	membrane depolarization[e]<br>++	membrane depolarization[e]<br>+	membrane depolarization[e]<br>+++	pore formation<br>(Matsuzaki, 1998)	pore formation<br>(Bolintineanu and Kaznessis, 2011**)**
Activity in apoplast extract[f] [10 µg/ml]	100%	50%	100%	n.d.	n.d.
Growth inhibition of bacteria/fungi on leaf surface [10 µg/ml]	*P. syringae pv syrimgae*<br>40%	*P. syringae pv syrimgae*<br>50%	*P. syringae pv syrimgae*<br>70%	*Alternaria solani*[g]<br>*Phytophtora infestans*[g]<br>80%	n.d.

[a] Hydrophobic amino acids in red letters, charged amino acids in blue letters, other are in grey.
[b] Peptide concentrations necessary to inhibit growth of the listed bacteria strains.

[c] Shown are the peptide concentrations leading to 25% hemoglobin release from human blood cells. >200 describes a slight hemolytic activity at 200 µg/ml but still below the above mentioned threshold, no value indicates no detectable hemolytic activity up to the highest concentration tested.
[d] Peptide concentration where protoplasts showing first signs of cytoplasmic degradation.
[e] Degree of membrane depolarization based on figure 3 and given with (+).
[f] Peptide activity in the presence of 10 µg/ml tomato apoplastic fluid.
[g] Tomato leaf disks were inoculated with peptide-treated conidia (*A. solani*) or sporangia (*P. infestans*).
n.d.: not determined

Bolintineanu, D.S., and Kaznessis, Y.N. 2011. Computational studies of protegrin antimicrobial peptides: a review. Peptides 32:188-201.

Matsuzaki, K. 1998. Magainins as paradigm for the mode of action of pore forming polypeptides. Biochim Biophys Acta 1376:391-400.

Ostberg, N., and Kaznessis, Y. 2005. Protegrin structure-activity relationships: using homology models of synthetic sequences to determine structural characteristics important for activity. Peptides 26:197-206.

Zasloff, M. 1987. Magainins, a class of antimicrobial peptides from Xenopus skin: isolation, characterization of two active forms, and partial cDNA sequence of a precursor. Proc Natl Acad Sci U S A 84:5449-5453.
